# Ventricular fibrillation mechanism and global fibrillatory organization are determined by gap junction coupling and fibrosis pattern

**DOI:** 10.1093/cvr/cvaa141

**Published:** 2020-05-13

**Authors:** Balvinder S Handa, Xinyang Li, Nicoleta Baxan, Caroline H Roney, Anastasia Shchendrygina, Catherine A Mansfield, Richard J Jabbour, David S Pitcher, Rasheda A Chowdhury, Nicholas S Peters, Fu Siong Ng

**Affiliations:** 1 National Heart & Lung Institute, Imperial College London, 4th Floor, ICTEM Building, 72 Du Cane Road, London W12 0NN, UK; 2 Biological Imaging Centre, Department of Medicine, Imperial College London, London, UK; 3 Division of Imaging Sciences and Bioengineering, King’s College London, London, UK

**Keywords:** Ventricular fibrillation, Fibrosis, Gap junctions, Rotational activity, Fibrillation, Mechanisms

## Abstract

**Aims:**

Conflicting data exist supporting differing mechanisms for sustaining ventricular fibrillation (VF), ranging from disorganized multiple-wavelet activation to organized rotational activities (RAs). Abnormal gap junction (GJ) coupling and fibrosis are important in initiation and maintenance of VF. We investigated whether differing ventricular fibrosis patterns and the degree of GJ coupling affected the underlying VF mechanism.

**Methods and results:**

Optical mapping of 65 Langendorff-perfused rat hearts was performed to study VF mechanisms in control hearts with acute GJ modulation, and separately in three differing chronic ventricular fibrosis models; compact fibrosis (CF), diffuse fibrosis (DiF), and patchy fibrosis (PF). VF dynamics were quantified with phase mapping and frequency dominance index (FDI) analysis, a power ratio of the highest amplitude dominant frequency in the cardiac frequency spectrum. Enhanced GJ coupling with rotigaptide (*n* = 10) progressively organized fibrillation in a concentration-dependent manner; increasing FDI (0 nM: 0.53 ± 0.04, 80 nM: 0.78 ± 0.03, *P* < 0.001), increasing RA-sustained VF time (0 nM: 44 ± 6%, 80 nM: 94 ± 2%, *P* < 0.001), and stabilized RAs (maximum rotations for an RA; 0 nM: 5.4 ± 0.5, 80 nM: 48.2 ± 12.3, *P* < 0.001). GJ uncoupling with carbenoxolone progressively disorganized VF; the FDI decreased (0 µM: 0.60 ± 0.05, 50 µM: 0.17 ± 0.03, *P* < 0.001) and RA-sustained VF time decreased (0 µM: 61 ± 9%, 50 µM: 3 ± 2%, *P* < 0.001). In CF, VF activity was disorganized and the RA-sustained VF time was the lowest (CF: 27 ± 7% vs. PF: 75 ± 5%, *P* < 0.001). Global fibrillatory organization measured by FDI was highest in PF (PF: 0.67 ± 0.05 vs. CF: 0.33 ± 0.03, *P* < 0.001). PF harboured the longest duration and most spatially stable RAs (patchy: 1411 ± 266 ms vs. compact: 354 ± 38 ms, *P* < 0.001). DiF (*n* = 11) exhibited an intermediately organized VF pattern, sustained by a combination of multiple-wavelets and short-lived RAs.

**Conclusion:**

The degree of GJ coupling and pattern of fibrosis influences the mechanism sustaining VF. There is a continuous spectrum of organization in VF, ranging between globally organized fibrillation sustained by stable RAs and disorganized, possibly multiple-wavelet driven fibrillation with no RAs.

## Graphical Abstract

**Figure:**
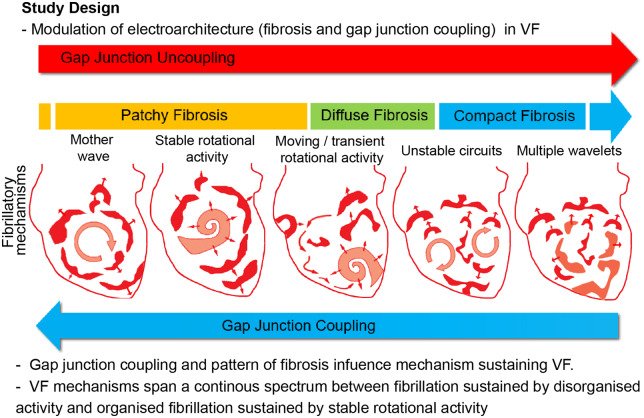

## 1. Introduction

Over the last five decades, multiple competing mechanisms have been implicated in sustaining ventricular fibrillation (VF). However, amongst experts in the field no consensus exists on a single unifying mechanism. Epicardial VF mapping studies in patients undergoing cardiac surgery on cardio-pulmonary bypass have shown evidence to support both disorderly perpetual multiple-wavelet activity in some patients and highly organized re-entrant waves sweeping the whole myocardium in others.^1^ These re-entrant wavefronts are often referred to as scroll waves, rotors, rotational activity (RA), or rotational drivers, and are characterized by pivoting around a phase singularity (PS) point and implicated in driving fibrillatory wavefronts. RAs have been mapped transmurally during VF in *ex vivo* perfused cardiomyopathic human hearts^2^ and more recently with non-invasive body surface mapping.^3^ It has been postulated that catheter-based ablation of regions localizing RAs may present a suitable therapeutic strategy in prevention of VF in VF survivors, however, the role and existence of RAs in patients remains highly controversial and largely unproven.

Whilst no consensus exists on a unifying fibrillatory mechanism, there is some evidence to suggest that differing degrees of cardiac organization and complexity exists in fibrillation. Optical mapping studies of coronary-perfused sheep ventricular slabs have previously shown a spectrum of VF complexity as characterized by dominant frequency (DF) analysis, although few reported instances of sustained RAs.^4^

Fibrosis and gap junction (GJ) remodelling are important substrates for the initiation and perpetuation of VF. High ventricular fibrosis burden post-myocardial infarction correlates with higher incidence of VF and ventricular tachycardia (VT).^5^ In limited perfused hearts studies, areas of high fibrosis anchor RAs in VF.^2^*In vitro* experiments with co-cultures of myocytes and myofibroblast have shown that an increase in the volume of myofibroblast relative to myocytes can increase complexity of propagation, increase wavefront fractionation, and reduce stability of re-entrant drivers that emerge.^6^ However, the link between the complexity of fibrillatory mechanism, RAs, and the degree and pattern of fibrosis in intact hearts is not clearly defined.

Cell–cell connectivity via GJs is important in electrical propagation between neighbouring cardiomyocytes. Abnormal expression and distribution of connexin43 has been implicated in increased vulnerability to developing ventricular tachyarrhythmias,^7^^,^^8^ whilst pre-treatment with GJ coupling enhancers reduces inducibility of VF^9^ in perfused hearts. However, the mechanism by which GJ coupling modulates underlying fibrillatory mechanisms is also uncertain.

In this study, fibrillatory dynamics were studied in *ex vivo* perfused rat hearts with optical mapping of transmembrane potentials in VF. We hypothesized that there is a continuous spectrum of fibrillatory organization and mechanisms, modulated by two important electroarchitectural components, namely the pattern and degree of fibrosis, and GJ coupling.

## 2. Methods

The detailed methods are in the Supplementary material online.

### 2.1 Ethical approval

This work was performed in accordance with standards set out in the UK Animals (Scientific Procedures) Act 1986, ARRIVE guidelines and was approved by Imperial College London Ethical Review Board under the project licence PEE7C76CD and PCA5EE967. All animal procedures conformed to the guidelines from Directive 2010/63/EU of the European Parliament on the protection of animals used for scientific purposes. For *ex vivo* studies requiring explantation of the heart, the rats were anaesthetized with 5% isoflurane (95% oxygen mix) in an induction chamber and euthanized with cervical dislocation.

### 2.2 Experimental protocols

VF optical mapping of transmembrane fluorescence was performed in 65 explanted Sprague-Dawley (SD, Charles River, Harlow, UK) rat hearts. The SD rats were 9–12 weeks old, weighing 250–300 g. VF mechanisms were studied with pharmacological GJ modulation of control hearts in VF with a GJ coupling enhancer, rotigaptide (RTG) (*n* = 10), GJ uncoupler, carbenoxolone (CBX) (*n* = 10), or control perfusate (*n* = 5). VF mechanisms were separately studied in a chronic 4-week ventricular fibrosis model with compact fibrosis (CF) (*n* = 11), diffuse fibrosis (DiF) (*n* = 11), and patchy fibrosis (PF) (*n* = 13). A sham surgery (*n* = 5) group was used as a control. In addition, to study the effect of enhanced GJ coupling in chronic fibrotic hearts, the DiF hearts above were also infused with RTG 80 nM after initial VF optical mapping. A schematic of study protocol is shown in Supplementary material online, *Figure S1*.

### 2.3 GJ coupling studies

A total of 25 control hearts were explanted and rapidly Langendorff-perfused with Krebs–Henseleit Buffer (KHB) solution (in mmol/L: NaCl 118.5, CaCl_2_ 1.85, KCl 4.5, glucose 11.1, NaHCO_3_ 25, MgSO_4_ 2.5, NaH_2_PO_4_ 1.4). VF optical mapping was performed at baseline and thereafter perfused with increasing concentrations of a GJ coupling enhancer, RTG (0–80nM, *n* = 10), a GJ uncoupler, CBX (0–50µM, *n* = 10), or control perfusate (*n* = 5).

### 2.4 Ventricular fibrosis

Three groups of ventricular fibrosis were generated in 35 rats. Separately, a sham surgical procedure was performed in five rats. All surgical recovery procedures were carried out with aseptic technique. The rats were first anaesthetized with 5% isoflurane (95% oxygen mix) inhalation in an induction chamber, intubated with a modified cannula, and ventilated using a Harvard rodent ventilator (MA, USA). Carprofen (5 mg/kg), enrofloxacin (5 mg/kg), vetergesic (0.05 mg/kg), and marcaine (0.5%) were administered subcutaneously as a single dose. Surgical permanent left anterior descending (LAD) artery ligation (*n* = 11) was performed to generate CF. Twenty minutes LAD territory ischaemia followed by reperfusion (*n* = 13) was used to generate PF. The methodology for inducing DiF was adopted from a study by Messroghli *et al.*,^10^ whereby an osmotic mini pump (Azlet 2ml4, CA, USA) pre-loaded to deliver 500 ng/kg/min of angiotensin (Abcam, Cambridge, UK) was implanted in the abdominal cavity. In the sham surgery group (*n* = 5), a suture was passed around the LAD without ligation. After surgery the rats were reviewed twice daily for adverse complications of the procedure (bleeding, infection, wound dehiscence) and pain was monitored by assessing behavioural changes, such as reduced feeding, loss of weight, ruffled coat, hunched posture, porphyrin staining, reduced mobility, ocular or nasal discharge, diarrhoea, and laboured breathing. Analgesia (Buprenorphine 0.05 mg/kg, subcutaneous administration) was given twice daily for the first 3 days, then reduced to once a day for another 4 days, and extended if needed beyond this period. After 4-week maturation, the hearts were explanted, Langendorff-perfused with KHB for VF optical mapping studies as previously described.^11^ Selected rats underwent *in vivo* cardiac magnetic resonance imaging (MRI) after anaesthetization with isoflurane (2.5%/95% oxygen mix) with late-gadolinium enhancement (LGE) prior to optical mapping.

### 2.5 VF optical mapping

VF was induced with provoked electrical stimulation using an extra stimulus protocol (8 beat S1 train, cycle length 100 ms, 2 mA, and successive earlier S2, S3, and S4 stimuli) or burst pacing protocol (20 beat train, 2 mA, cycle length 40–100 ms) in all hearts from an implanted electrode. All hearts were treated with a potassium channel opener, Pinacidil (30 µM) to aid VF maintenance prior to optical mapping studies. Propensity to sustained VF induction was scored on an arrhythmia provocation scoring system (described in Supplementary material online). The excitation-contraction uncoupler, blebbistatin (Tocris Bio-Sciences, Cambridge, UK) was infused through a side port at a loading dose of 30 µM, followed by a maintenance concentration of 10 µM set up to recirculate in the perfusate. The hearts were stained with a voltage-sensitive dye (40 µl of 5 mg/mL RH237 in dimethyl sulfoxide; Thermo-Fisher, MA, USA) given as a slow bolus through the side port. A custom made 128 × 80 pixel complementary metal-oxide-semiconductor camera (Cairn, Faversham, UK) was used to record the optical fluorescence signals (Supplementary material online, *Figure S2*). All recordings were of the left ventricular (LV) anterior epicardial surface and 10 seconds in duration with a sampling rate of 1000 frames/s.

### 2.6 VF phase analysis

Optical fluorescence data were filtered and phase processed as previously described.^12^ All raw optical fluorescence signals were processed in MATLAB R2018 (MathWorks, MA, USA) using custom made scripts. The data were first filtered using methodology and code adapted from the Efimov laboratory mapping toolbox.^13^ Briefly, the signals were spatially filtered by binning in a 3-by-3 pixel matrix, high-frequency noise was removed with 0–100 Hz low pass filter, baseline drift was removed, and signals normalized. The filtered VF optical fluorescence data were analysed with a custom made MATLAB (R2018, MathWorks) fibrillation analysis script.^14^ The methodology for phase analysis has been previously described.^12^^,^^15^ Briefly, each pixel of optical fluorescence data was tagged for the minima and maxima and filtered to remove small amplitude fluctuations in the signals and fitted to a cubic spline to subtract the average of the minima and maxima splines to generate a zero mean. The real and imaginary parts of the Hilbert transform of this zero-mean signal were plotted in the phase plane and the phase angle calculated from this. A phase map of VF at each sampled time point was constructed and PSs / RAs tagged using our algorithm (Supplementary material online, *Figure S2*).

### 2.7 VF data analysis

Prior to induction of sustained VF, baseline conduction velocity (CV) and APD90 data in response to pacing at differing cycle lengths for the fibrosis and GJ groups were obtained as shown in Supplementary material online, *Figure S4*. From the phase processed data, the edge of each wavefront was tracked in a 9 × 9 pixel window and RAs characterized with quantification of rotations, duration, rotational frequency, and meander. Furthermore, the total number of RAs/s and total duration of all RAs/s of fibrillatory recordings were calculated. A minimum two-rotation filter was used to threshold and define a significant RA and to construct RA heat maps. The methodology and parameters for wavefront tracking and RA characterization, such as wavefront length, RA spatial gap, and temporal gap were determined by sensitivity testing as previously described.^14^ The path of the longest duration RAs (defined as those with at least >5 rotations) was tracked and meandering expressed as the centre shift per RA rotation. Centre shift was calculated as √(*x*^2^ + *y*^2^), whereby *x* and *y* are number of pixels of displacement in the *x* and *y* plane from the initiation point to termination point of the RA. The centre shift was normalized to the number of rotations for a given RA by dividing the centre shift by number of rotations for a given RA and expressed as pixels of centre shift displacement per rotation.

### 2.8 Frequency dominance index

Cardiac fibrillatory organization was measured using the frequency dominance index (FDI). The FDI is defined as the proportion of area occupied by the largest organized DF area in the global fibrillatory spectrum divided by the total area of all regions with a defined DF (Supplementary material online, *Figure S3*). The methodology for calculating FDI is similar to an index proposed by Berenfeld *et al.*^16^, domain density, which quantifies the number of DF domains per cm^2^. The methodology for calculating DF has been previously described in detail.^12^

### 2.9 Statistical analysis

The Kolmogorov–Smirnov normality tests were applied to the data. When distribution was normal, Student’s *t*-test or ANOVA (*post hoc* Bonferroni) statistical analyses were performed. *n* represents the number of experiment performed with rat hearts. For repeated measures with a single variable (i.e. GJ coupling experiments with differing concentrations), a repeated measures ANOVA (*post hoc* Bonferroni) was applied. A *P*-value <0.05 was considered statistically significant. Statistical analysis was performed with a commercially available software package GraphPad Prism 5.0. All values are expressed as mean ± standard error of mean or median with inter-quartile range (25–75%).

## 3. Results

### 3.1 The degree of GJ coupling alters the VF electrocardiogram

**Figure 1:**
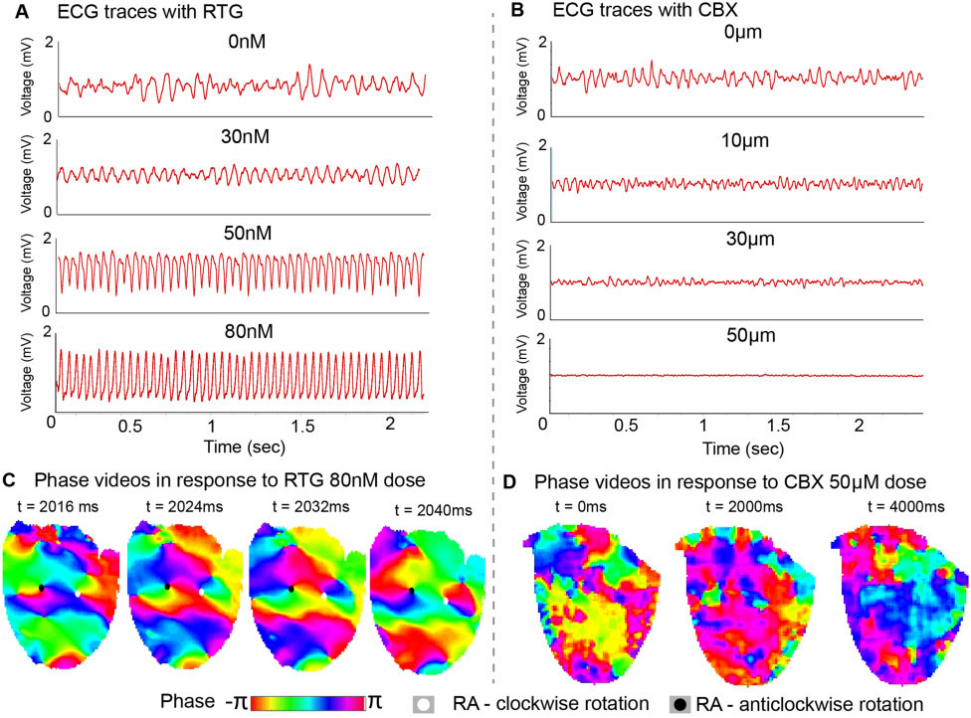
Enhanced GJ coupling regularizes VF to VT. Representative ECGs in response to (*A*) RTG (0–80 nM) with progressive regularization of VF to VT and (*B*) CBX (0–50 µM) with progressive VF disorganization on ECG. (*C*) Representative phase maps with tracked RAs in VF in response to RTG 80 nM showing existence of spatially stable RAs. (*D*) Representative phase maps in VF in response to CBX 50 µM showing disorganized myocardial activation only.

Initially to establish whether GJ coupling altered fibrillatory mechanisms, changes in the global field electrocardiogram (ECG) morphology were studied in response to different degrees of GJ coupling. Pharmacological GJ modulation altered the periodicity and global organization of VF on the ECG. Enhancing GJ coupling with RTG regularized VF in a concentration-dependent manner, progressively organizing VF to VT at the 80 nM concentration (*Figure 1A*). The DF power spectral density (PSD) analysis of the ECG traces showed a number of DFs in the global fibrillation spectrum at baseline, and as GJ coupling enhanced with RTG this organized to a single DF with a high PSD (Supplementary material online, *Figure S5A*). In contrast, GJ uncoupling with CBX progressively reduced the amplitude of the VF ECG and disorganized VF in a concentration-dependent manner (*Figure 1B*). The DF PSD analysis of the ECG traces showed no well-defined DF as GJ uncoupling increased (Supplementary material online, *Figure S5B*). To examine these changes in the ECG VF morphology further, we studied the underlying mechanism in detail with phase analysis of high-resolution optical mapping recordings of VF activation patterns. Enhanced GJ coupling with high-dose RTG (80 nM) demonstrated VF sustained by spatially stable long-duration RAs on phase mapping (*Figure 1C*), however, at high degrees of GJ uncoupling (with 30 and 50 µM CBX), there was no discernible activation pattern or periodicity identifiable on phase mapping (*Figure 1D*).

### 3.2 GJ modulation alters the underlying mechanism of VF

**Figure 2:**
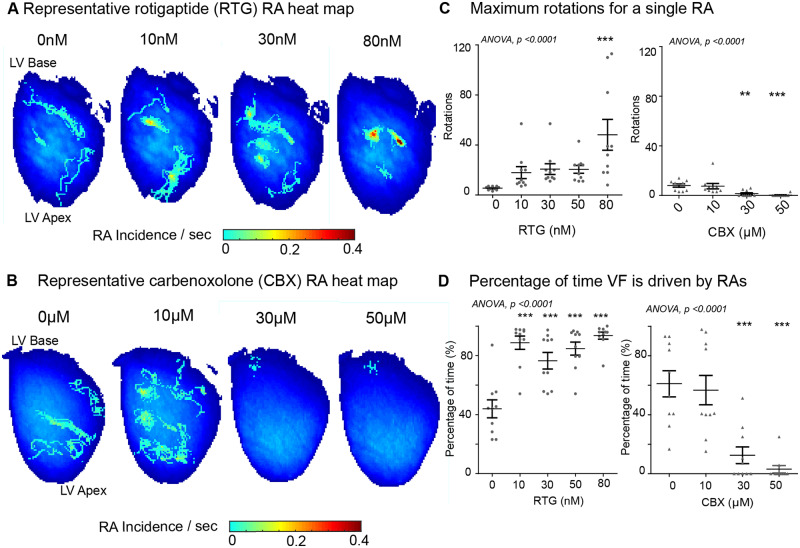
The degree of GJ coupling influences the stability of RAs. Representative RA heat map in (*A*) RTG- and (*B*) CBX-treated hearts in VF. (*C*) Progressive increase in maximum rotations for a single RA in RTG group in comparison to decrease with CBX. (*D*) Increase in percentage of VF time driven by RAs at increasing RTG concentrations comparative to decrease with CBX. Data from RTG (*n* = 10) and CBX (*n* = 10) hearts. Statistical analysis with repeated measures ANOVA, *post hoc* Bonferroni, *P*-values are in comparison to baseline, **P < 0.01 and ***P < 0.001.

A systematic quantitative study of phase processed recordings of VF activation in response to different degrees of GJ coupling was performed. Enhancing GJ coupling with RTG stabilized RAs in a concentration-dependent manner, localizing them to discrete areas in VF (*Figure 2A*). In contrast, uncoupling GJs with CBX destabilized RAs in VF, increased their meander, and shifted the mechanism towards disorganized activity (*Figure 2B*). In the RTG-treated hearts, there was a significant increase in the maximum rotations tracked for a single RA (baseline: 5.4 ± 0.5 rotations vs. 80 nM: 48.2 ± 12.3 rotations, *P* < 0.001), whilst with CBX there was a progressive decrease with increased GJ uncoupling (baseline: 8.0 ± 1.3; 30 µM: 1.5 ± 0.8, *P* < 0.01, 50 µM: 0.3 ± 0.3, *P* < 0.001, *Figure 2C*), and at higher degrees of uncoupling, infrequent RAs were short-lived, but largely non-existent. The percentage of time VF was sustained by RAs increased 2.1-fold in the RTG-treated group (baseline: 44 ± 6%, 80 nM: 94 ± 2%, *P* < 0.001) and decreased significantly with CBX (baseline: 61 ± 9%, 30 µM: 13 ± 6%, 50 µM: 3 ± 2%, *P* < 0.001, *Figure 2D*).

**Figure 3:**
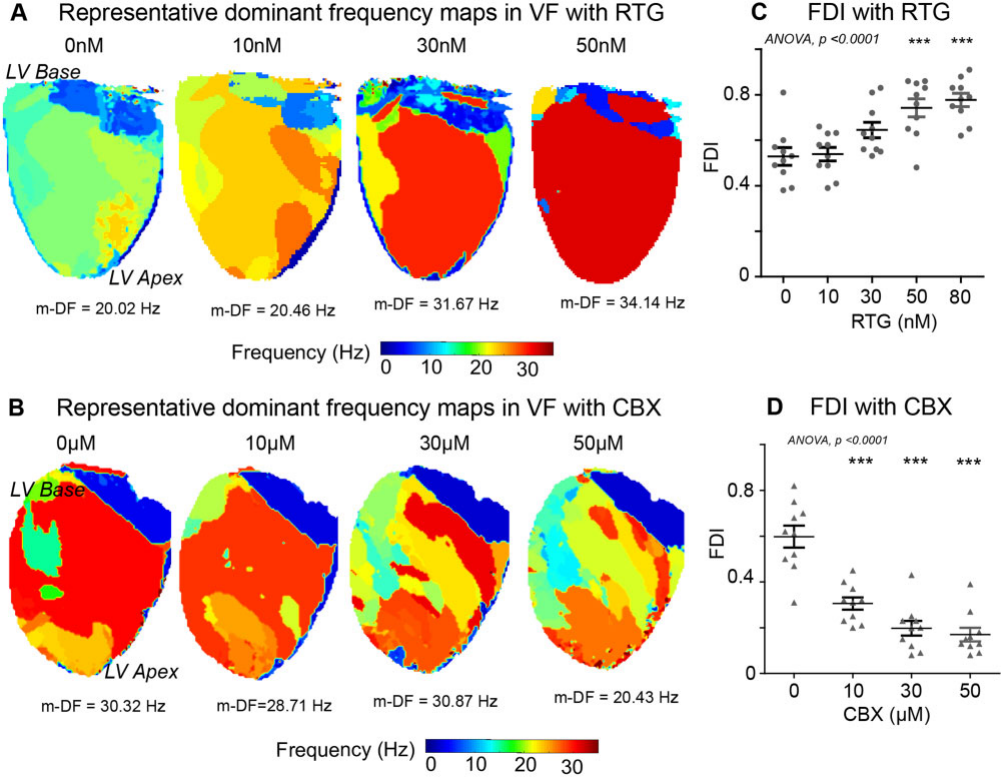
Enhanced GJ coupling increases the global fibrillatory organization. Representative DF maps in response to (*A*) RTG and (*B*) CBX in VF. The DF of the largest organized region or the m-DF is indicated below in hertz (Hz). (*C*) A graph showing an increase in the FDI in RTG-treated hearts in comparison to (*D*) decrease with CBX. Data from RTG (*n* = 10) and CBX (*n* = 10) hearts. Statistical analysis with repeated measures ANOVA, *post hoc* Bonferroni, *P*-values are in comparison to baseline, ***P < 0.001. m-DF, maximal DF.

In addition to phase analysis, VF activation was studied with analysis of the global frequency spectrum of fibrillatory activation. Enhanced GJ coupling with RTG progressively organized multiple DFs in the fibrillation spectrum to only one predominant DF sustaining fibrillation (*Figure 3A* and Supplementary material online, *Figure S6A*). In contrast, GJ uncoupling with CBX progressively disorganized fibrillation, with multiple DFs sustaining VF with no clearly organized region of maximum DF (*Figure 3B* and Supplementary material online, *Figure S6B*). The FDI, a measure of global organization derived from DF analysis, increased with RTG (baseline: 0.53 ± 0.04, 50 nM: 0.74 ± 0.04, 80 nM: 0.78 ± 0.03, *P* < 0.001, *Figure 3C*) and decreased with CBX (baseline: 0.60 ± 0.05, 30 µM: 0.20 ± 0.03, 50 µM: 0.17 ± 0.03, *P* < 0.001, *Figure 3D*). In the control group, no significant changes were observed in fibrillatory mechanism over a time period exceeding GJ coupling experiments in the percentage of VF time sustained by RAs, stability of RAs or changes in the underlying DF spectrum of fibrillation (Supplementary material online, *Figures S7 and S8*). Other parameters for measuring stability of RAs, including maximum duration, average rotations, total duration/s, and total RAs/s were significantly higher with enhanced GJ coupling in the RTG group compared to baseline (Supplementary material online, *Figure S9*) and reduced progressively with increasing CBX-mediated GJ uncoupling (Supplementary material online, *Figure S10*).

### 3.3 The pattern of ventricular fibrosis alters VF ECG

**Figure 4:**
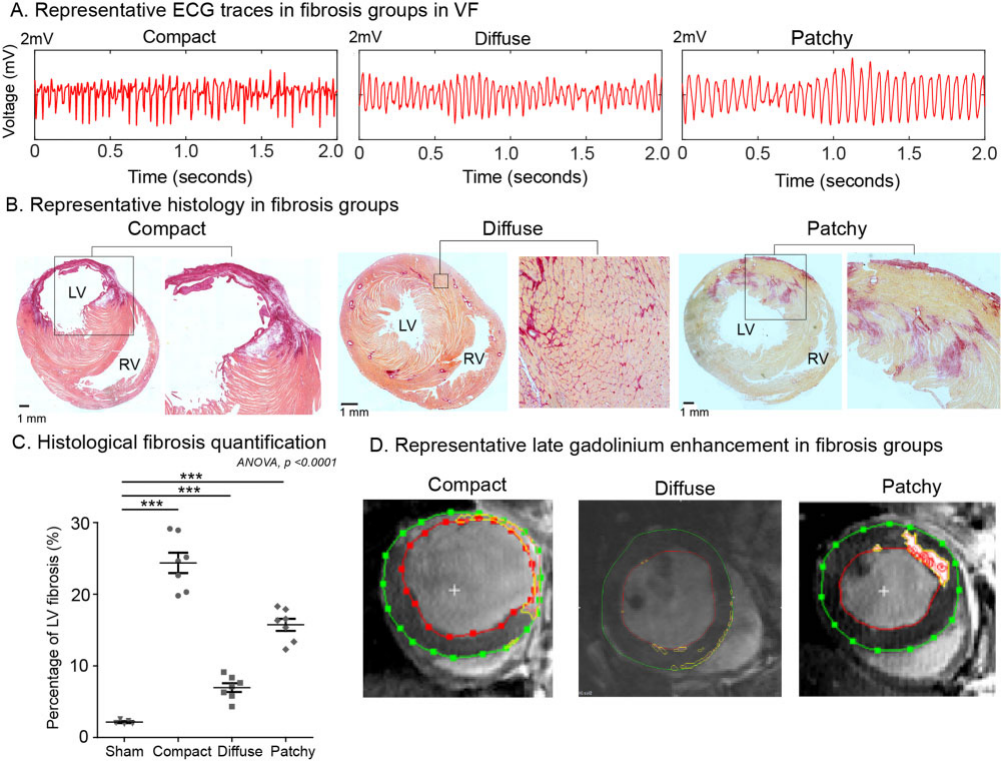
Validation of differing ventricular fibrosis models. (*A*) Representative ECG traces in differing fibrosis models in VF. (*B*) Representative mid-LV axial histological sections of fibrotic areas (dark red) and normal tissue (orange to pink), scale—black bar corresponds to 1 mm. (*C*) Histological LV fibrosis quantification. (*D*) Representative mid-LV short-axis slices with LGE. Data in (*C*) from select hearts; sham (*n* = 4), CF (*n* = 7), DiF (*n* = 7), and PF (*n* = 7) hearts. Statistical analysis with ANOVA *post hoc* Bonferroni multiple comparisons test, ***P < 0.001.

We next studied whether physical heterogeneity in ventricular tissue generated through fibrosis caused a similar spectrum of changes in global VF organization and mechanisms. As with GJ coupling experiments, during validation of the fibrosis models, the periodicity and global organization of VF on the ECG was found to vary between the differing fibrosis groups, with PF hearts demonstrating a relatively stable activation frequency and CF the most variable (*Figure 4A*). ECG DF PSD analysis showed that there was a lack of a well-defined DF in the CF group. The PF group demonstrated a single large DF with a high PSD value. The DiF group showed a DF with an intermediate PSD value and a small number of DFs clustered around it (Supplementary material online, *Figure S11*). Histological validation of the differing fibrosis models showed both differing quantities and complexity of fibrosis patterns. CF demonstrating dense confluent fibrosis with thinned LV anterior wall and the highest quantity of fibrosis, DiF demonstrating interlacing interstitial fibrosis and PF demonstrating islands of fibrosis amongst normal myocardial tissue (*Figure 4B and C*). The propensity to induction of sustained VF varied between fibrosis groups and was the highest in the CF group (Supplementary material online, *Figure S12*). Whilst MRI with LGE reliably detected compact and patchy ventricular fibrosis, the sensitivity was poor for detection of diffuse fibrosis (*Figure 4D* and Supplementary material online, *Figure S13*). Action potential duration (APD) showed no significant differences between the differing fibrosis groups in remote regions. However, APD dispersion was increased in the infarct border zone regions in the LAD-ligation/CF group and ischaemia-reperfusion/PF model (Supplementary material online, *Figure S14*).

### 3.4 Pattern of fibrosis is a key determinant of underlying fibrillatory mechanism

**Figure 5:**
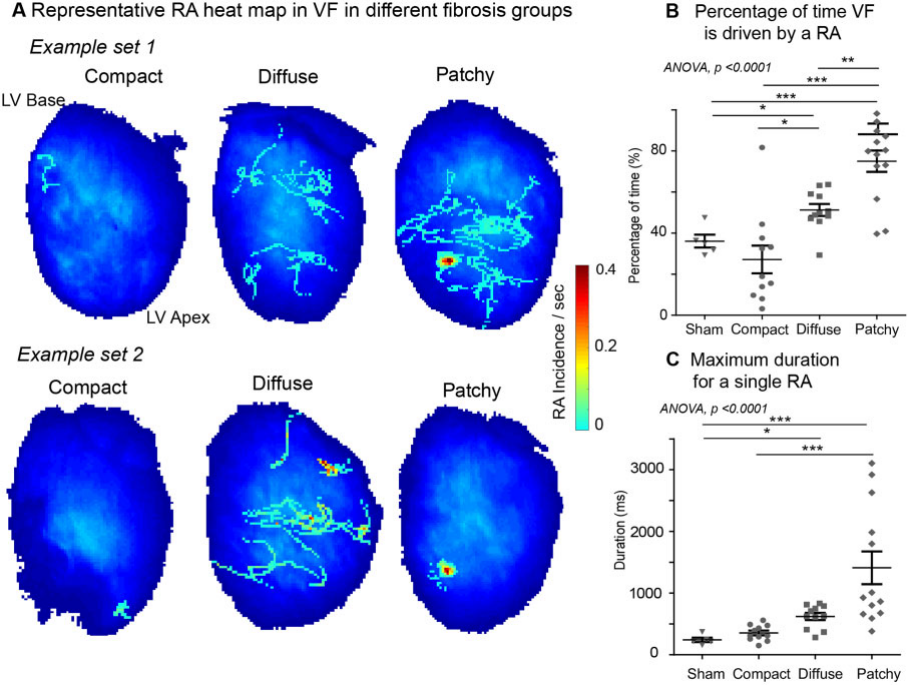
The pattern of fibrosis influences the stability of RAs in fibrillation. (*A*) Representative RA heat maps for differing fibrosis models; CF, DiF, and PF hearts in VF. (*B*) Percentage of VF time driven by RAs and (C) maximum duration for a single RA in the PF group is higher in comparison to sham surgery, CF and DiF groups. Data from sham surgery (*n* = 5), CF (*n* = 11), DiF (*n* = 11), and PF (*n* = 13) hearts. Statistical analysis with ANOVA, *post hoc* Bonferroni multiple comparisons test, *P < 0.05, **P < 0.01, ***P < 0.001.

VF was sustained predominantly by disorganized activity in CF, and was most globally organized in PF, with the DiF group exhibiting an intermediate VF phenotype. Percentage of time VF was sustained by RAs was highest in PF; sham: 36 ± 7% vs. patchy: 75 ± 5%, *P* < 0.001, compact: 27 ± 7% vs. patchy, *P* < 0.001, and diffuse: 51 ± 3% vs. patchy, *P* < 0.001 (*Figure 5A and B*). PF frequently sustained spatially stable RAs localized to discrete areas, DiF harboured transient and meandering RAs, whilst in the CF group stable RAs were rarely detected (*Figure 5A*). The stability of RAs was influenced by fibrosis patterns, with PF harbouring most stable longest duration RAs; sham: 242 ± 80 ms vs. patchy: 1411 ± 266 ms, *P* < 0.01 and diffuse: 620 ± 57 ms vs. patchy, *P* < 0.01 (*Figure 5C*). Numerous other parameters for stability of RAs, including maximum rotations, average rotations, and total RAs/s were all highest in the PF group (Supplementary material online, *Figure S15*).

**Figure 6:**
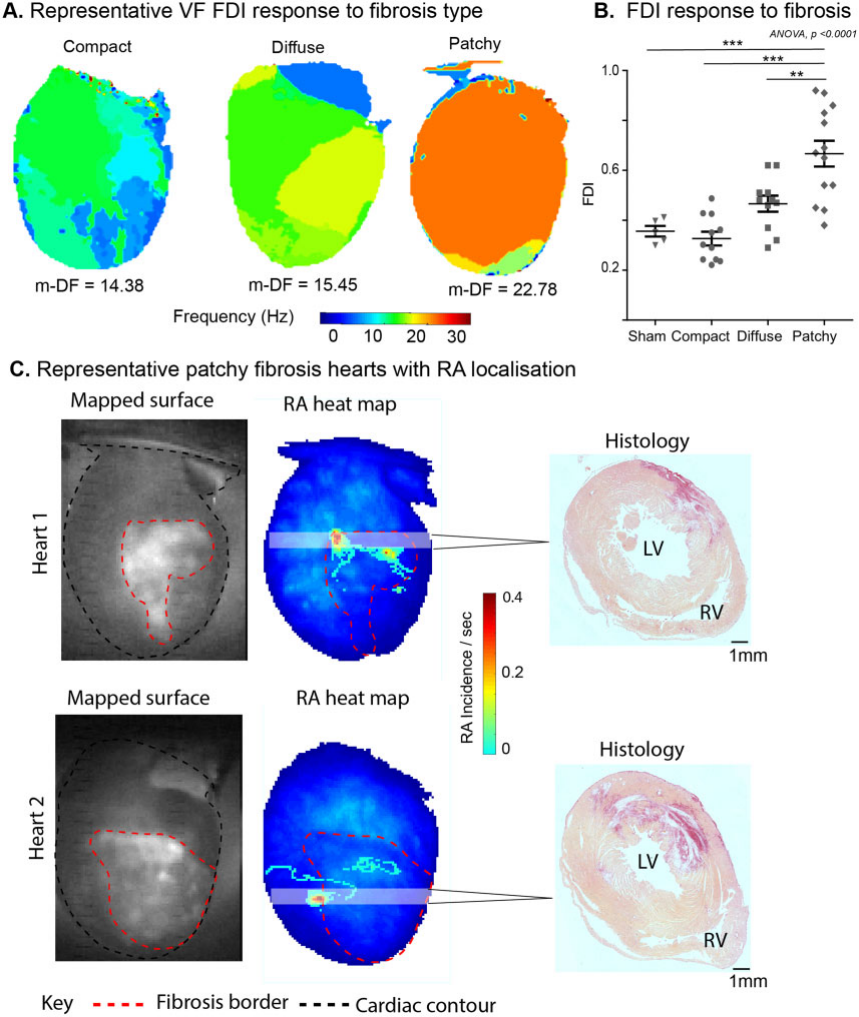
PF anchors RAs and sustains the most globally organized fibrillation. (*A*) Representative DF maps in differing fibrosis groups; CF, DiF, and PF hearts in VF. The DF of the largest organized region or the m-DF is indicated below in hertz (Hz). (*B*) A graph showing the highest FDI in the PF group in comparison to DiF and CF. (*C*) Two representative PF hearts showing the mapped surface with fibrosis border in view (left), phase processed RA heat maps (middle), and corresponding histological cross-section from the shaded region (pink, pale yellow = normal myocardium, dark red = fibrosis, scale—black bar = 1 mm). Data from sham surgery (*n* = 5), CF (*n* = 11), DiF (*n* = 11), and PF (*n* = 13) hearts. Statistical analysis with ANOVA, *post hoc* Bonferroni multiple comparisons test, **P < 0.01, ***P < 0.001. m-DF, maximal DF.

The DF map in PF showed that VF was predominantly sustained by a single large amplitude DF, comparative to the other two groups where multiple DF were seen in VF (*Figure 6A* and Supplementary material online, *Figure S16*). FDI was highest in the PF group and lowest in the CF group, with DiF displaying intermediate FDI values (sham: 0.36 ± 0.02 vs. patchy: 0.67 ± 0.05, *P* < 0.001, compact: 0.33 ± 0.03 vs. patchy, *P* < 0.001) (*Figure 6B*). Furthermore, in the PF group, the areas harbouring stable RAs frequently localized to regions where fibrosis interfaced with viable myocardium (*Figure 6C*).

### 3.5 Enhanced GJ coupling and PF reduced meander of RAs

**Figure 7:**
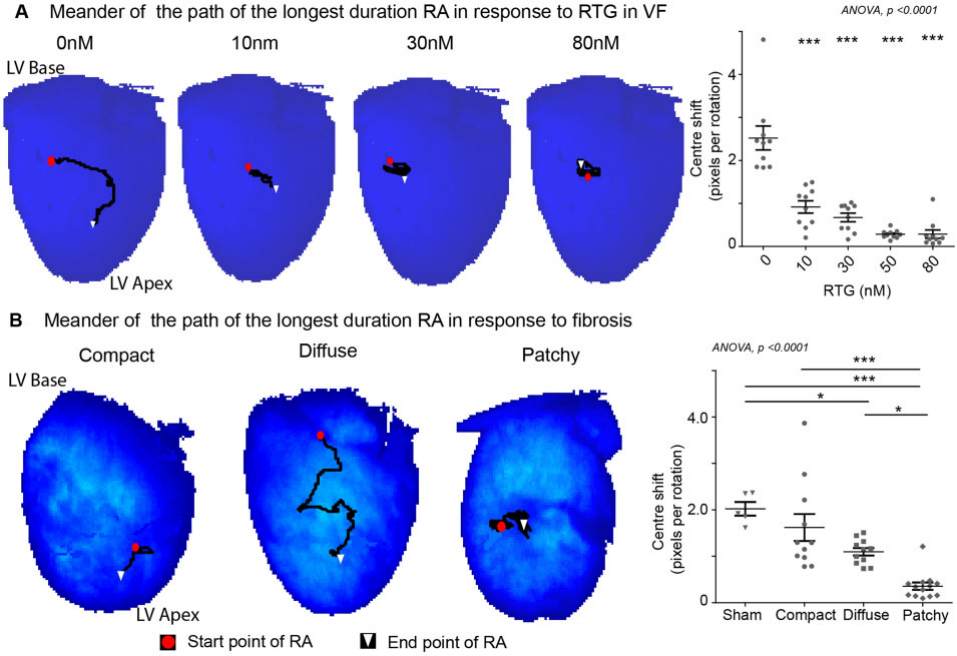
The meander of RAs is lowest in PF and reduces with enhanced GJ coupling. Representative tracked paths of the longest duration RA (threshold—>5 rotations) in (*A*) RTG-treated group and (*B*) differing fibrosis groups; CF, DiF, and PF (left) and the corresponding centre shift from initiation to termination site over time (right). Data from RTG (*n* = 10) treated hearts in (*A*) and sham surgery (*n* = 5), CF (*n* = 11), DiF (*n* = 11), and PF (*n* = 13) hearts in (*B*). Statistical analysis with repeated measures ANOVA, *post hoc* Bonferroni, *P*-values are in comparison to baseline in (*A*) and ANOVA, *post hoc* Bonferroni multiple comparisons test in (*B*). *P < 0.05 and ***P < 0.001.

We studied the spatial stability of the longest duration RAs (>5 rotation threshold) in VF by tracking their path and quantifying the meander with centre shift measures. Enhanced GJ coupling with RTG stabilized RAs to discrete regions and reduced their meander. The centre shift per rotation of longest duration RAs reduced progressively with RTG is a concentration-dependent manner (baseline: 2.52 ± 0.28, 50 nM: 0.28 ± 0.03, 80 nM: 0.29 ± 0.10 pixels per rotation, *P* < 0.001) (*Figure 7A*). Similarly, with fibrosis, despite PF harbouring the longest duration RAs, they localized to a small area, whereas in the DiF group RAs meandered significantly. Centre shift in the PF groups was significantly lower than DiF (patchy: 0.36 ± 0.08 vs. diffuse: 1.10 ± 0.08 pixels per rotation, *P* < 0.05, *Figure 7B*). The CF group harboured only a few RAs that met the five-rotation threshold, however, they demonstrated a higher degree of meander comparative to other fibrosis groups (patchy vs. compact: 1.62 ± 0.30 pixels per rotation, *P* < 0.001).

### 3.6 Enhanced GJ coupling organized and terminated VF in chronic fibrosis

After determining that VF mechanism were influenced by both GJ coupling and fibrosis, we next studied the effects of maximally enhancing GJ coupling (with 80 nM RTG dose) in VF in hearts with chronic DiF. Maximally enhancing GJ coupling in VF in chronic DiF hearts resulted in VF regularizing in periodicity and organizing to VT before terminating in 5/11 hearts (Supplementary material online, *Figure S17A*). Enhanced GJ coupling with RTG increased FDI significantly (baseline: 0.47 ± 0.03; 80 nM: 0.72 ± 0.03) and altered the VF mechanism from multiple DFs to a predominant single DF prior to termination. However, equally, the mean DF across the mapped surface was lower after RTG infusion and it is uncertain whether this change leads to VF termination (Supplementary material online, *Figure S17B*).

## 4. Discussion

This study shows that VF is sustained by a continuous spectrum of mechanisms, which range from globally organized fibrillation sustained by stable RAs to disorganized fibrillation without stable RAs. Fibrillatory mechanisms are critically influenced by the changes in the underlying electroarchitectural components, specifically, fibrosis patterns and the degree of GJ coupling. We propose this may provide an explanation for the differing mechanisms previously reported in sustaining fibrillation. We also demonstrate an intrinsic link between temporally and spatially stable RAs with globally organized fibrillation.

### 4.1 Spectrum of fibrillation organization and mechanisms

VF mapping in at-risk patient groups is highly challenging, and the understanding of mechanisms sustaining VF remains poor. Limited insight into VF mechanisms comes from animal studies, where conflicting data have emerged implicating multiple wavelets, focal activation, anatomical and functional re-entrant drivers.^17^ The potential existence of stable RAs in VF has led some investigators to explore mechanism-guided ablation as a therapeutic option.^3^ In this study, we provide an explanation for the differing mechanisms reported and show that only some forms of VF have stable RAs. Data from this study shows that VF is sustained by a continuous spectrum of global fibrillatory organization and that the level of fibrillatory organization relates specifically to the mechanism sustaining it. Globally organized fibrillation was sustained by a predominant large area of a single DF with stable RAs localized to a small area. Globally disorganized fibrillation, however, had no clearly organized region of maximum DF and was sustained by disorganized activity with no stable RAs. Whilst in between the two ends of the organizational scale, fibrillation was sustained by a mixture of disorganized activity and transient unstable RAs, with multiple small areas with a defined DF. A previous study by Zaitsev *et al.*^4^ also demonstrated a spectrum of complexity of VF in coronary-perfused normal sheep ventricular slabs from endocardial and epicardial mapping studies, although the complexity of the arrhythmia was attributed to the rate of VF (as measured by the mean DF of the recording). Whilst the FDI quantified global fibrillatory organization in this study, the sites of the highest DF were not considered. The role of the highest DF site and the spatial distribution of DFs in fibrillation is not entirely clear. In atrial fibrillation (AF) ablation of regions with the highest DF, previously thought to be driving the fibrillatory mechanism, was shown not to improve outcomes.^18^ However, ablation of the maximal DF regions reduces intra-atrial DF gradients and homogenizes the spatial DF distribution in patients who eventually become AF free.^19^

Whilst our work was in a VF model, it may be reasonable to extrapolate our mechanistic findings with caution to AF, where mechanisms remain intensely debated. Whilst the atria and ventricle differ significantly in anatomy, geometry, APD profiles, and the role of certain critical initiation sites, similar mechanisms have been shown to maintain fibrillation in both. As such, common mechanistic considerations may be given to the concept of ‘myocardial fibrillation’. In some AF optical mapping studies, stable RAs are rarely seen and 98% of fibrillation has been shown to be sustained by wavelets resulting from the breakup of more organized high frequency organized waves.^20^ To the contrary, the Haïssaguerre group reported presence of RAs in 82% of AF patients, some exhibiting up to eight rotations and numerous instances of acute AF termination with RA targeted ablation.^21^ In contrast, the endocardial–epicardial hypothesis frames fibrillation as a largely disorganized phenomenon of continuous and chaotic focal breakthroughs that propagate transmurally with few connection and continuous regeneration.^22^ In this work, we have systemically shown the presence of a spectrum of fibrillatory organization, its relationship to the mechanism sustaining it, and the underlying electroarchitecture. This may explain these discrepancies in findings in AF, although further work in experimental atrial models is needed to corroborate the findings here.

### 4.2 GJ coupling determines VF organization and mechanism

The degree of GJ coupling was found to determine the degree of fibrillatory organization along a continuous spectrum between globally organized fibrillation sustained by stable RAs and disorganized fibrillation with no RAs in this study. Reduction in GJ function and connexin expression, specifically connexin43, has been implicated in the initiation and maintenance of VF.^23^ Our results provide strong, direct evidence for its role by demonstrating a concentration-dependent change in fibrillatory mechanisms with the degree of GJ coupling.

GJ uncoupling with CBX reduces CV and increases conduction heterogeneity without affecting ionic currents or APD.^24^ CV slowing is implicated in arrhythmia susceptibility, however, its impact on the mechanism of fibrillation is unknown. In the atria, CV heterogeneity and slowing has been implicated in stabilizing sites of RA.^25^ To the contrary, we found that high degrees of uncoupling increased tortuosity of conduction paths and a more disorganized form of fibrillation developed. Similar increases in complexity of fibrillatory activity in cell culture experiments using a GJ uncoupler have been reported.^26^ Zlochiver *et al.*^6^ demonstrated that low and heterocellular coupling through silencing of connexin43 in myocyte and myofibroblast cell cultures reduced CV and complexity of wavefront propagation whilst destabilizing RAs.

GJ coupling was enhanced in this work with RTG, which phosphorylates connexin43 serine residues. Enhanced GJ coupling reduces the inducibility of ventricular tachyarrhythmias;^27^ and reduces energy needed for VF cardioversion in pre-treated perfused hearts.^28^ GJ remodelling has also been described in AF and implicated in increased vulnerability to developing AF.^29^ As with VF, pre-treatment with GJ coupling enhancers also reduces inducibility of AF.^30^ In a VT/VF mechanistic study in a perfused rabbit model prepared with cryoablation, GJ modulation fundamentally influenced stability of RAs.^31^ Whilst the mechanisms defined differed from ours, possibly due to differences in species, dosing and their use of an excitation-contraction uncoupler that alters ionic current (2,3-butanedione monoxime), enhanced GJ coupling was also found to terminate VF as per our finding and GJ uncoupling perpetuated it. Myocardial fibrosis is associated with tortuous and slow conduction and disorganized connexin43 distribution.^7^ Enhanced GJ coupling is known to normalize CV slowing physiologically stressed states such as ischaemia and disease remodelling.^32^ Myocardial metabolic demand during VF is also greatly increased beyond that of a normally beating heat,^33^ and VF itself may drive a relatively ischaemic state. This may provide an explanation for the progressive organization of fibrillation, stabilization of RAs to discrete regions, and eventual termination seen with enhanced GJ coupling in fibrotic hearts.

### 4.3 The role of cardiac fibrosis in VF mechanisms

We systematically demonstrated that the fibrosis pattern alters the fibrillatory organization and its mechanism of sustaining VF. The role of cardiac fibrosis is well established, and there is a correlation between the quantity of fibrosis and VF propensity,^34^ although it is unclear how differing fibrotic patterns affect the underlying fibrillatory mechanisms. Limited evidence exists to show that RAs localize to areas of greater fibrosis in VF in perfused cardiomyopathic hearts^2^ and to low voltage areas, a surrogate for fibrosis.^35^ Similarly in AF, RAs localized to areas of MRI identified complex fibrotic patterns in a limited study of perfused human left atria.^36^ In keeping with our finding, *in silico* studies have suggested that anisotropy and CV heterogeneity in PF regions stabilize and anchor RAs,^37^ whereas in DiF, RAs are less stable and meander randomly.^38^ Our results provide direct experimental evidence showing a direct link between different fibrotic patterns and specific fibrillation mechanisms. CF models exhibited a disorganized fibrillatory pattern and this fibrosis pattern did not harbour any stable RAs, possibly due to the dense non-conductive nature of CF being unable to anchor or sustain stable re-entrant circuits. The sham surgery group, like CF, harboured few RAs and had a VF phenotype predominantly sustained by disorganized activity. In the PF groups, areas harbouring the most stable RAs were frequently localized to areas of fibrosis interspersed amongst viable myocardium. These findings suggest that structural and electrophysiological heterogeneity resulting from the interface between complex fibrosis and viable myocardium, such as in PF and DiF, is a critical substrate for anchoring stable RAs and sustaining a globally organized fibrillation.

### 4.4 Clinical implications

Understanding the organization and underlying mechanism of fibrillation can facilitate a patient-tailored treatment approach towards VF prevention in VF survivors. Organized fibrillation sustained by spatiotemporally stable drivers may be considered for targeted ablation. Disorganized fibrillation dynamics may be better suited for conventional pharmacotherapy. These findings may hold some relevance in AF in selecting patient for pharmacotherapy based on a disorganized mechanism instead of ablation. However, more work is needed in AF to corroborate findings of this study and caution should be exercised in extrapolating the findings here.

### 4.5 Limitations

Two-dimensional epicardial data from this study may not reflect the transmural mechanism in three-dimensional cardiac tissue. Similarly, optical mapping was non-panoramic and limited to the LV anterior wall in the field of view of the camera and thus fibrillatory activity from the right ventricle was not recorded. Meandering RA’s originating or terminating outside the mapped field of view may have influenced the quantitative analysis of RA dynamics in this study. A number of other factors, including remodelling of ionic currents and calcium handing abnormalities, which are important known determinants of the fibrillatory mechanisms were outside the scope of this study and were not investigated. In specific, changes in regional inwardly rectifying potassium currents^39^^,^^40^ and calcium currents^41^ have been shown to influence the stability of RAs, and the pattern and fragmentation of wavebreaks in myocardial fibrillation. We did not specifically quantify GJ coupling at different concentrations of pharmacological modulation, though data exist to demonstrate effects on GJs at doses utilized in this work. Furthermore, rat cardiac action potential profile and APD differs significantly from human, and further large animal studies are needed to corroborate the findings of this study.

## 5. Conclusion

In summary, we demonstrated that the underlying fibrillatory organization and its mechanism is influenced by GJ coupling and the pattern of fibrosis, two important electroarchitectural components of the arrhythmic substrate. A continuous spectrum of global fibrillatory organization and its specific relationship to the mechanism sustaining VF was shown in this study, and we propose that this provides an explanation to reconcile hitherto apparently conflicting reports on fibrillation mechanisms in the literature. Fibrillatory mechanisms exist along a continuum between globally organized fibrillation sustained by stable RAs, intermediately organized with a mix of unstable meandering RAs and disorganized activity, through to disorganized with no RAs. Characterizing global fibrillatory organization and mechanism sustaining VF may facilitate a patient-tailored treatment approach towards VF prevention in VF survivors.

## Data availability

The data underlying this article will be shared on reasonable request to the corresponding author.

## Supplementary material

Supplementary material is available at *Cardiovascular Research* online.

## Authors’ contributions

B.S.H. conducted all the experiments, generated the ventricular fibrosis models, performed data analysis, and wrote the manuscript. X.L. wrote the codes for quantitative fibrillation analysis and aided with data analysis. N.B. performed the cardiac MRI. A.S. conducted some of the cardiac histology. C.H.R. wrote the original phase analysis code used in data analysis. C.M. and R.J.J. assisted with the recovery surgical procedures. D.S.P. and R.A.C. assisted with data analysis and optical mapping. N.S.P. provided co-supervision of the project and was a co-applicant on the funding application. F.S.N. conceptualized the study design, co-wrote the funding application, supervised the project, and reviewed the manuscript.

**Conflict of interest:** none declared.

## Funding

This work was supported by the British Heart Foundation (RG/16/3/32175 and PG/16/17/32069). F.S.N. was also supported by the National Institute for Health Research (NIHR) Imperial Biomedical Research Centre and an NIHR Clinical Lectureship (CL-2011-21-001). C.H.R. acknowledges a Medical Research Council Skills Development Fellowship (MR/S015086/1). N.B. is supported by the BHF Centre for Regenerative Medicine (RM/17/1/33377).

**Translational perspective**

Multiple competing mechanisms have been proposed for sustaining ventricular fibrillation (VF). We reframed conflicting mechanisms reported in sustaining fibrillation and defined them as part of a continuum of varying global organization, with some sustained by stable rotational activities (RAs). The underlying cardiac electroarchitecture, namely gap junction coupling and fibrosis, were important determinants of the VF mechanism. Characterizing the VF mechanism and its relationship to the cardiac electroarchitecture may facilitate a patient-tailored treatment approach towards VF prevention in VF survivors. Organized fibrillation sustained by stable RAs could be considered for targeted ablation. Disorganized fibrillation dynamics may be better suited for conventional pharmacotherapy.

## Supplementary Material

cvaa141_Supplementary_Data

## Notes

**Time for primary review: 28 days**
