# Supplementary material for: Ventricular fibrillation mechanism and global fibrillatory organization are determined by gap junction coupling and fibrosis pattern
Source: Cardiovasc Res. 2020 May 13;117(4):1078–90. doi: 10.1093/cvr/cvaa141 (PMC7983010; doi:10.1093/cvr/cvaa141)

**Supplementary Materials:** **Ventricular fibrillation mechanism and global fibrillatory organisation are determined by gap junction coupling and fibrosis pattern**

Balvinder S. Handa, BSc, MRCP^1^, Xinyang Li, PhD^1^, Nicoleta Baxan, PhD^2^, Caroline Roney, PhD^3^, Anastasia Shchendrygina, MSc^1^, Catherine A. Mansfield, PhD^1^, Richard Jabbour, BSc, MRCP^1^, David Pitcher, PhD^1^, Rasheda A Chowdhury, PhD^1^, Nicholas S. Peters, FRCP, MD^1^, Fu Siong Ng, MRCP, PhD^1^

^1^ National Heart & Lung Institute, Imperial College London, United Kingdom

^2^ Biological Imaging Centre, Department of Medicine, Imperial College London, London

^3^ Division of Imaging Sciences and Bioengineering, King's College London, United Kingdom

**Conflict of interests:** None declared.

Correspondence to:

Dr Fu Siong Ng,

4th Floor, ICTEM Building,

Imperial College London,

72 Du Cane Road,

W12 0NN, United Kingdom.

E-mail: [f.ng@imperial.ac.uk](mailto:f.ng@imperial.ac.uk)

Phone: +44 (0)20 7594 3614

**Supplementary Methods**

*Ethical Approval.* This work was performed in accordance with standards set out in the United Kingdom Animals (Scientific Procedures) Act 1986, in accordance to ARRIVE guidelines and was approved by Imperial College London Ethical Review Board under the project license PEE7C76CD and PCA5EE967. All animal procedures conformed to the guidelines from Directive 2010/63/EU of the European Parliament on the protection of animals used for scientific purposes. For ex vivo studies requiring explantation of the heart, the rats were anaesthetised with 5% isoflurane (95% oxygen mix) in an induction chamber and euthanised with cervical dislocation.

*Experimental Protocols.* VF optical mapping of transmembrane fluorescence was performed in 65 explanted Sprague-Dawley (SD, Charles River, Harlow, UK) rat hearts. The SD rats were 9-12 weeks old, weighing 250-300g. VF mechanisms were studied in a chronic 4-week ventricular fibrosis model with compact (n=11), diffuse (n=11), patchy (n=13) fibrosis and sham surgery (n=5), and separately with pharmacological GJ modulation of control hearts with control (n=5), rotigaptide (n=10) or carbenoxolone (n=10) in VF.

*Myocardial infarction surgery to generate compact and patchy fibrosis.* The model for generating compact fibrosis from coronary artery ligation and patchy fibrosis from ischaemia-reperfusion through temporary coronary artery ligation have been previously described[1–4]. 39 SD rats in total underwent myocardial infarction (MI) surgery, of these 29 survived to 4 weeks. For the MI surgery rats weighing 250-300g were first anaesthetized with 5% isoflurane (95% oxygen mix) inhalation in an induction chamber and intubated with a 16G modified cannula. The rats were ventilated using a Harvard rodent ventilator (Massachusetts, USA). Carprofen (5mg/kg), enrofloxacin (5mg/kg), vetergesic (0.05mg/kg) and marcaine (0.5%) was administered subcutaneously as a single dose. The chest wall was cleaned using betadine and draped. A left sided thoracotomy was performed at the level of the 4th intercostal space with a small oblique incision. The subcutaneous layer was blunt dissected, pectoralis major and minor retracted to expose the intercostal muscles. A small incision was then made in the 4th intercostal space and the heart was exposed. The pericardium was gently stripped and the thymus was clamped in position to stabilise the heart. In the compact fibrosis group a permanent Prolene 7-0 suture was placed at the mid left anterior descending artery (LAD) around 3mm below the lower border of the left atrium at a depth of 1-2mm. In the patchy fibrosis group, in contrast, a small slip-knot suture was placed instead at the same mid LAD level for 20 minutes only. In both groups, infarction was confirmed by pallor and akinesis of the anterior wall of the LV and apex. In the patchy fibrosis group, the slip knot was then released and reperfusion confirmed with visual inspection of the LV wall. The incisions was closed with absorbable sutures (Vicryl 4-0). The rats were extubated and recovered in a warm chamber until ambulant. The infarcts were matured for four weeks prior to the experiment. The sham surgery group (n=5) underwent the same process as myocardial infarction surgery group, with the exception of the Prolene 7-0 suture being passed around the LAD only without ligation.

*Angiotensin infusion to generate diffuse fibrosis.* In this group 11 out of 13 rats survived to 4 weeks.The model for inducing diffuse ventricular fibrosis without a heart failure phenotype in this study was adopted from that previously described by Messroghli et al. [5]. The rats (250g-300g) were anaesthetized with 5% isoflurane (95% oxygen mix) inhalation in an induction chamber and kept under general anaesthetic using a Bains-coaxial breathing apparatus with 1 – 2.5% isoflurane (97.5 – 99% oxygen mix). The epigastric region was cleaned with betadine, draped and vetergesic (0.05mg/kg) and marcaine (0.5%) was administered subcutaneously as a single dose. A small vertical incision was made and the subcutaneous layers, fascia and linea alba was blunt dissected in between the abdominal muscles until access was gained to the intraabdominal cavity. An osmotic mini pump (Azlet 2ml4, California, USA) pre-loaded to deliver 500ng/kg/min of angiotensin (Abcam, Cambridge, UK) was implanted in the abdominal cavity in right iliac fossa. The incisions was closed with absorbable sutures (Vicryl 4-0) and the animals recovered in a warm chamber until ambulant. The infusion was delivered for a total of 4 weeks before optical mapping studies.

Post recovery surgery, all rats were closely monitored twice daily for adverse complications of the procedure (bleeding, infection, wound dehiscence) and pain was monitored by assessing behavioural changes (such as reduced feeding, loss of weight, ruffled coat, hunched posture, porphyrin staining, reduced mobility, ocular or nasal discharge, diarrhoea and laboured breathing. Twice daily analgesia (Buprenorphine 0.05 mg/kg, subcutaneous administation) was given for the first 3 days, then reduced to once a day for 4 days and extended if needed beyond this period.

**Cardiac MRI**

In the cardiac fibrosis group, select rats; sham (n=3), compact (n=5), patchy (n=5) and diffuse (n=5) underwent cardiac MRI for characterization of fibrosis prior to optical mapping studies. Rats were anesthetised and maintained under inhalational anesthesia via a nose cone (2.5% isoflurane/medical oxygen). Vital signs and ECG was continuously monitored. All MRI scans were performed on a pre-clinical 9.4 T scanner (94/20 USR Bruker BioSpec; Bruker Biospin, Ettlingen, Germany) housed at the Biological Imaging Centre, Imperial College London equipped with a volume transmit quadrature coil combined with an actively decoupled rat heart array receiver. Data were acquired with Paravision 6.01 (Bruker, BioSpin). For localisation of the heart, low-resolution ECG and respiratory triggered gradient echo scout scans were acquired in axial, sagittal and coronal orientations followed by pseudo two- and four-chamber gradient echo scans. Multi slice T_1_ mapping was performed right after gadolinium contrast injection (0.5 mmol/kg Gadovist, Bayer, USA,) using a look-locker inversion recovery sequence with 25 inversion times (TI) which followed an adiabatic global inversion time. All inversions were ECG triggered to allow images to be acquired at the same part of the cardiac cycle, with the TI points restricted to multiples of the R-R interval. Multi slice late gadolinium enhancement (LGE) data were acquired using an inversion recovery gradient echo sequence with a single TI point and flip angle of 90°. The TI was selected from the respective T_1_ maps to effectively null the healthy myocardium and to provide the best contrast enhancement of the area of infarction. Fibrosis was quantified in conformity to the American Heart Association (AHA) 17 segment model by tracking LGE for each LV axial slice using the in-built function in the open source software Segment (version 2.2, Medviso AB, Lund, Sweden). The following acquisition parameters were used: TE = 1.4 ms, TR_s_ = 3.85 ms, FOV = (38 ×38) mm^2^, matrix size = 180 × 180, spatial resolution (210×210) µm^2^, 1 mm slice thickness, 9 slices, scan time: 3 min /slice.

*Langendorff Perfusion.* After a 4-week maturation period the chronic fibrosis hearts were explanted, heparinized, and rapidly perfused ex-vivo on a Langendorff apparatus with Krebs-Henseleit solution (in mmol/l: NaCl 118.5, CaCl2 1.85, KCl 4.5, glucose 11.1, NaHCO3 25, MgSO4 2.5, NaH2PO4 1.4) gassed with 95% O2/5% CO2 at 37 °C ± 0.5 °C and pH 7.35 ± 0.05. A 10 minute stabilisation period was allowed during which the flow rate (10-15ml/min), temperature (37 °C ± 0.5 °C) and perfusion pressure (90-100mmHg) through the aorta was maintained.

*ECG recordings.* Electrograms were recorded as field ECGs with two electrodes sited in the perfusate surrounding the perfused hearts using Labchart 7.0 (AD Instruments, Sydney, Australia). Electrodes were connected to a Bioamplifier and a PowerLab data acquisition system (AD Instruments, Sydney, Australia).

*VF induction with programmed electrical stimulation (PES).* VF was induced with provoked electrical stimulation using an extra stimulus protocol (8 beat S1 train, cycle length 100ms, 2mA, and successive earlier S2, S3 and S4 stimuli) or burst pacing protocol (2mA, cycle length 40-100ms, 20 beat train) using a silver electrode placed at the bases of the left ventricles with a MicroPace system (Micropace EP, Santa Ana, USA). VF was induced in a stepwise approach and inducibility was additionally scored using an arrhythmia provocation score in all hearts. All hearts were treated with a potassium channel opener, Pinacidil (30µM) to aid maintenance of VF prior to optical mapping studies. VF was allowed to perpetuate for 3 minutes prior to optical mapping studies. Induced VF was continuously sustained in all Pinacidil treated hearts in this study and did not self-terminate prior to fibrosis and GJ coupling experiments described below.

*Arrhythmia provocation scoring.* The propensity to VF was tested with increasingly pro-arrhythmic stimuli in the fibrosis hearts and scored on a custom scale adapted from previously described methods [6,7]. An arrhythmia provocation score (APS) was allocated between 1 to 7 based on the response to PES, with 7 corresponding to the highest propensity to sustained VF and 1 to the lowest. The APS for sustained positive VF induction were as follow: 7- a single extra stimulus (S1 and S2), 6 - two extra stimuli (S1, S2 and S3), 5- three extra stimuli (S1, S2, S3, S4), 4- burst pacing (100-70ms cycle length), 3- burst pacing (40-70ms cycle length), 2 – extrastimuli protocol (S1, S2,S3,S4) with aid of pincadil 30µM pre-treatment, 1- burst pacing (40-70ms) with aid of pinacidil 30µM pre-treatment. Of note, all hearts received Pinacidil 30µM prior to VF optical mapping after arrhythmia provocation scoring if not already received.

*Gap junction modulation.* To characterise the effect of GJ modulation on VF mechanisms 25 control Sprague-Dawley (SD) rats weighing 250-300g were euthanized and the hearts were explanted. The hearts were rapidly perfused, stabilised, Langendorff perfused and had VF induced and sustained as described above for the fibrosis groups. After induction of VF, either a GJ coupling enhancer, rotigaptide (RTG, 0-80nM,n=10) or a GJ coupling blocker (CBX, carbenoxolone, 0-50uM,n=10) was added to the recirculating perfusate incrementally at increasing doses for optical mapping studies. A perfusion period of 5 minutes was allowed between each dose change prior to optical mapping. In the control group (n=5), 1ml of the Krebs-Henseleit perfusate was added at time intervals over 30 minutes. This time period exceeded the time course of GJ coupling experiments. Separately, to study the effect of enhanced GJ coupling in chronically fibrotic hearts, the diffuse fibrosis hearts (n=11) above were infused with the maximal 80nM rotigaptide dose after undergoing VF optical mapping. **Supplementary Figure 1** shows the study design figuratively.

*Optical mapping.* After the stabilisation period on the Langendorff apparatus, an excitation-contraction uncoupler, blebbistatin (Tocris Bio-Sciences, Cambridge UK) was infused through a side port at a loading dose of 30µM, followed by a maintenance concentration of 10µM set up to recirculate in the perfusate. The hearts were stained with a voltage-sensitive dye (40µl of 5mg/ml RH237 in dimethyl sulfoxide; Thermo-Fisher, Massachusetts, USA) given as a slow bolus through the side port. The epicardial surface was excited using multiple light emitting diodes (530nm) and emitted light was detected by a custom made 128 x 80 pixel complementary metal-oxide-semiconductor (CMOS) camera (Cairns, Faversham, UK). Signals were recorded by pacing from an electrode placed below the left atrium in anterior wall of the LV and/or after induction of ventricular fibrillation with programmed electrical stimulation. Each optical mapping recording was 10 seconds in length with a frame rate of 1000 frames / second.

*Histology.* A select number of fibrotic hearts (sham – 4/5, compact – 7/11, diffuse 7/11, patchy 7/13) underwent histological characterisation after optical mapping studies. The samples were fixed in 4% formaldehyde and thereafter embedded in paraffin-wax. 10 µm thickness sections were cut at intervals of 1mm from the apex to the base of the ventricles and stained with Picrosirus red stain (Abcam, Cambridge, UK) for characterizing fibrosis. Digital images were acquired with high-resolution scanning of slides using a widefield HWF1 Zeiss AxioObserver microscope. The images were scanned using the Zeiss proprietary Zen2012 Acquisition software (Carl Zeiss AG, Germany). The images were analysed in Fiji64 software (ImageJ, open-source). In the diffuse and patchy fibrosis group, the LV was isolated (and the RV excluded), thereafter the fibrotic tissue was thresholded from normal myocardium, and the percentage of fibrosis expressed as fibrotic area divided by total LV tissue area x 100%. In the compact fibrosis group, the LV was isolated as before, to account for thinning of LV anterior wall, the line method was used, whereby percentage fibrosis was calculated from total fibrosis epicardial and endocardial length divided by normal myocardium total epicardial and endocardial length x 100%.

**Data Analysis**

*Phase Mapping.* All raw optical fluorescence signals were processed in MATLAB R2018 (MathWorks, Massachusetts, USA) using custom made scripts. The data was first filtered using methodology and code adapted from the Efimov laboratory mapping toolbox. Briefly, the signals were spatially filtered by binning in a 3-by-3 pixel matrix, high frequency noise was removed with 0-100Hz low pass filter, baseline drift was removed, and signals normalised. These techniques have been previously described in detail [8]. The filtered VF optical fluorescence data was analysed with a custom made MATLAB (R2018, MathWorks, Massachusetts, USA) fibrillation analysis script [9]. The methodology for this has been previously described [10,11]. Briefly, each pixel of optical fluorescence data was tagged for the minima and maxima and filtered to remove small amplitude fluctuations in the signals and fitted to a cubic spline to subtract the average of the minima and maxima splines to generate a zero mean. The real and imaginary parts of the Hilbert transform of this zero-mean signal were plotted in the phase plane and the phase angle calculated from this. A phase map of VF at each sampled time point was constructed and PS tagged using our algorithm **(Supplementary Figure 2)**.

The edge of each wavefront was tracked in a 9x9 pixel window and rotational activities (RAs) characterised with quantification of rotations, duration, rotational frequency and meander. Furthermore, the total number of RAs per second and total duration of all RAs per second of fibrillatory recordings was calculated. A minimum 2 rotation filter was used to threshold and define a significant rotational activity and to construct phase singularity heats maps. Parameters for wavefront tracking and RA characterisation, such as wavefront length, RA spatial gap and temporal gap were determined by sensitivity testing. The path of the longest duration rotational activities (defined as those with at least > 5 rotations) were tracked and expressed as the centre shift per RA rotation. Centre shift was calculated as √(x^2^+y^2^), whereby x and y are number of pixels of displacement in the x and y plane from the initiation point to termination point of the RA. The centre shift was further normalised to the number of rotations for a given RA by dividing the centre shift by number of rotations for a given RA, and expressed as pixels of centre shift displacement per rotation.

*Dominant Frequency (DF) Analysis*

The methodology for calculating DF has been previously described in detail [11]. Briefly, the DF of a time-series signal is defined as the frequency with highest energy in the power spectrum. The time-domain signal was transformed into the frequency domain using a discrete Fourier transform (or fast Fourier transform) and the relative power spectrum density of each frequency was calculated. For a spectrum X(f), the dominant frequency is defined as the frequency of highest amplitude by the equation:

$$DF=\text{arg }\max_{f}X\left( f \right)$$

In this study DF analysis was applied to global field electrocardiograms and single pixel optical fluorescence data.

*Frequency dominance index.*

The FDI calculates the total level of global organisation by analysing all the DFs from all the signals within a fibrillating ventricle. The FDI is defined as the largest organised DF area in the global fibrillatory spectrum relative to the total area of all regions with a defined DF (**Supplementary Figure 3**). FDI is expressed as a proportion. For instance, a dominant frequency signal of 14 Hz in 60% of the mapped areas would give a FDI of 0.6

*Statistical Analysis*

The Kolmogorov-Smirnov normality tests were applied to the data. When distribution was normal, Student’s t-test, or ANOVA (post hoc Bonferroni) statistical analyses were performed. n represents the number of experiment performed with rat hearts. For repeated measures with a single variable (i.e. gap junction coupling experiments with differing concentrations), a repeated measures ANOVA (*post hoc* Bonferonni) was applied. A P-value <0.05 was considered statistically significant. Statistical analysis was performed with a commercially available software package GraphPad Prism 5.0. All values are expressed as mean ± standard error of mean or or median (interquartile range),

**Supplementary Results**

Prior to induction of sustained VF, baseline conduction velocity and APD90 data paced at differing cycle lengths for the differing fibrosis groups and gap junction groups was obtained as shown in **Supplementary Figure 4**.

**Effects of GJ modulation on fibrillation signal dominant frequencies**

**Supplementary Figure 5** shows the DF power spectral density (PSD) of the ECG traces in Figure 1A and 1B. At baseline VF, there are a number of DFs in the global fibrillation spectrum, as GJ coupling enhances with rotigaptide, fibrillation organised to VT, driven by a single DF with a high PSD. GJ uncoupling with carbenoxolone had the opposite effect on VF ECG response and at high degrees of GJ uncoupling, there was no well-defined DF present.

**Supplementary Figure 6A** shows the representative DF traces corresponding to DF maps of VF optical mapping in Figure 3 of the main text. With enhanced GJ coupling mediated by rotigaptide, the frequency spectrum of the fibrillatory signal becomes organised and increasingly uniform, whereby at the maximum rotigaptide concentration one large amplitude DF is driving VF (**Supplementary Figure 6A**). Whereas, with GJ uncoupling mediated by carbenoxolone, the frequency spectrum disorganises in fibrillation and at maximum carbenoxolone concentrations multiple dominant frequencies are driving the overall fibrillation signal (**Supplementary Figure 6B**).

**Fibrillatory mechanisms over time in control group remained unchanged**

In the control treated group, over a time course greater than the GJ coupling experiments, the underlying mechanism of fibrillation remained stable. **Supplementary Figure 7A** shows a representative heart in the control group where the DF spectrum of fibrillation remained similar over time and there was no significant changes in the FDI (baseline: 0.42 ± 0.02, 10 mins: 0.39±0.02, 20 mins: 0.43 ±0.03, 30 mins: 0.41 ± 0.02, **Supplementary Figure 7B**). The RA heat map showed a similar pattern of short-lived and spatially dispersed RAs over recorded time intervals (**Supplementary Figure 7C)**. The percentage of time VF was driven by RAs showed no significant change (baseline: 41 ± 2%, 10 mins: 39 ± 1%, 20 mins: 44 ± 2%, 30 mins: 39 ± 2%, **Supplementary Figure 7D**). Similarly, a number of other metrics of RA stability showed no significant change over time in comparison to baseline in VF **(Figure 8).**

**Effects of enhanced GJ coupling on rotational activity stability**

Rotigaptide mediated enhanced GJ coupling improved stability of rotational activities by a number of different metrics. The total maximum duration for a single RA increased 5.2 fold between baseline and maximal concentration (Baseline: 244 ± 17.5 ms, 80nM: 1626 ± 359.9 ms, p<0.001, **Supplementary Figure 9A**). Similarly, the average rotations for all RAs increased significantly (Baseline: 2.3 ± 0.1 rotation, 80nM: 5.7 ± 0.4 rotations, p<0.001, **Supplementary Figure 9B**). The total number of RAs and their total duration per second of fibrillation also increased significantly compared to baseline with enhanced GJ coupling **(Supplementary Figure 9C, 9D).**

**Effects of GJ uncoupling on rotational activity stability**

Carbenoxolone mediated GJ uncoupling destabilised rotational activities by a number of different metrics. At increasing carbenoxolone concentrations fibrillation increasingly driven by disorganised multiple wavelet mechanism rather than stable RAs. The total maximum duration for a single RA decreased significantly between baseline and maximal concentration (Baseline: 300 ± 36.0 ms, 50µM: 7.1 ± 7.1 ms, p<0.001, **Supplementary Figure 10A**). Similarly, the average rotations for all RAs decreased (Baseline: 3.8 ± 0.4 rotations, 50µM: 0.36 ± 0.3 rotations, p<0.001, **Supplementary Figure 10B**). The total number of RAs and their total duration per second of fibrillation also decreased compared to baseline with increasing GJ uncoupling (**Supplementary Figure 10C, 10D**).

**Dominant frequency spectrum of baseline VF in fibrosis**

DF analysis of the VF ECG traces in differing fibrosis (corresponding to ECGs in Figure 4A) showed differing response. **Supplementary Figure 11** shows that in the compact fibrosis group there was a lack of a well-defined DF. The patchy fibrosis group demonstrated a single large DF with a high PSD value. The diffuse fibrosis group showed a DF with an intermediate PSD value and a small number of DFs clustered around it.

**Arrhythmia provocation**

The propensity for induction and perpetuation of VF was the highest in the compact fibrosis group. The APS in compact fibrosis was statistically higher than sham and other fibrosis groups (sham: 1.0±0 versus compact: 4.6±0.6, p<0.001, diffuse: 1.8±0.2 vs compact, p<0.01, **Supplementary Figure 12**). In the GJ experiments, where all hearts in the control and GJ experiment groups were structurally normal, there was no statistical difference between groups in APS (control: 1.2±0.2, RTG: 1.1±0.1, CBX: 1.3±0.2, p = 0.57, results not enclosed).

**Characterisation of ventricular fibrosis**

LGE MRI was able to differentially characterise differing fibrosis models. In both the patchy and compact fibrosis groups, the fibrotic regions showed effective gadolinium uptake and LGE quantification of fibrosis quantity was similar to histology, however, gadolinium uptake in the diffuse fibrosis group was comparatively poor and fibrosis was underestimated (**Supplementary** **Figure 13**).

The APD90 showed no significant difference between the differing fibrosis group in the remote or the border zone regions (**Supplementary Figure 14**). The APD90 dispersion was statistically greater in the fibrosis border zone compared to the remote region in both the permanent MI (remote: 2.73 ± 0.33 ms versus fibrosis border zone: 7.02 ± 0.48 ms, p<0.001) and IR group (remote: 7.76 ± 0.3 3ms versus fibrosis border one 7.88 ± 0.46ms, p<0.001).

**Effects of differing fibrosis patterns on rotational activity stability**

The pattern of fibrosis influenced the stability of RAs, with patchy fibrosis harbouring the most stable RAs. The total maximum rotations for a single RA was the highest in the patchy fibrosis group (sham: 5.4±0.7 versus patchy: 34.7±5.4, p<0.001, **Supplementary Figure 15A**). In the compact fibrosis group RAs we unstable and exhibited a low number of average rotations (Compact: 2.4 ± 0.3 rotations versus patchy: 5.2 ± 0.6 rotations, p<0.001**, Supplementary Figure 15B**). The total number of RAs and their total duration per second was also the highest in the patchy fibrosis group. Amongst all metrics of RA stability, diffuse fibrosis harboured intermediate values between compact and patchy fibrosis (**Supplementary Figure 15C, 15D**).

**Frequency dominance index response in differing fibrosis groups**

**Supplementary Figure 16A** shows that DF histograms in patchy fibrosis demonstrated that VF was predominantly sustained by a single large amplitude DF, comparative to the other two groups where multiple DF were seen in VF in the global fibrillatory spectrum (corresponding to DF maps in Figure 6A,).

**Enhanced GJ coupling organised and terminated VF in chronic fibrosis**

The effects of maximally enhancing GJ coupling (with 80nM RTG dose) in VF in hearts with chronic diffuse fibrosis was studied. Maximally enhancing GJ coupling in VF in chronic diffuse ventricular fibrosis hearts resulted in VF regularising in periodicity and organising to VT before terminating in 5/11 hearts **(Supplementary Figure 17A).** Enhanced GJ coupling with RTG increased FDI significantly (Baseline: 0.47±0.03; 80nM: 0.72±0.03) and altered the VF mechanism from multiple DFs to a predominant single DF prior to termination **(Supplementary Figure 17B)**.

**References for Supplement**

[1] Pfeffer MA, Pfeffer JM, Fishbein MC, Fletcher PJ, Spadaro J, Kloner RA, Braunwald E. Myocardial infarct size and ventricular function in rats. Circ Res 1979;44(4):503–12.

[2] Pfeffer MA, Braunwald E. Ventricular remodeling after myocardial infarction. Experimental observations and clinical implications. Circulation 1990;81(4):1161–72.

[3] Deloche A, Fabiani JN, Camilleri JP, Relland J, Joseph D, Carpentier A, Dubost C. The effect of coronary artery reperfusion on the extent of myocardial infarction. Am Heart J 1977;93(3):358–66.

[4] Hale SL, Kloner RA. Left ventricular topographic alterations in the completely healed rat infarct caused by early and late coronary artery reperfusion. Am Heart J 1988;116(6):1508–13.

[5] Messroghli DR, Nordmeyer S, Dietrich T, Dirsch O, Kaschina E, Savvatis K, O h-Ici D, Klein C, Berger F, Kuehne T. Circ Cardiovasc Imaging 2011;4(6):636–40.

[6] Ng FS, Kalindjian JM, Cooper SA, Chowdhury RA, Patel PM, Dupont E, Lyon AR, Peters NS. Enhancement of Gap Junction Function During Acute Myocardial Infarction Modifies Healing and Reduces Late Ventricular Arrhythmia Susceptibility. JACC Clin Electrophysiol 2016;2(5):574–82.

[7] Bélichard P, Savard P, Cardinal R, Nadeau R, Gosselin H, Paradis P, Rouleau JL. Markedly different effects on ventricular remodelling result in a decrease in inducibility of ventricular arrhythmias. J Am Coll Cardiol 1994;23(2):505–513.

[8] Laughner JI, Ng FS, Sulkin MS, Arthur RM, Efimov IR. Processing and analysis of cardiac optical mapping data obtained with potentiometric dyes. Am J Physiol Heart Circ Physiol 2012;303:H753-65.

[9] Li X, Roney CH, Handa BS, Chowdhury RA, Niederer SA, Peters NS, Ng FS. Standardised Framework for Quantitative Analysis of Fibrillation Dynamics. Sci Rep. 2019 Nov 13;9(1):16671.

[10] Roney CH, Ng FS, Debney MT, Eichhorn C, Nachiappan A, Chowdhury RA, Qureshi NA, Cantwell CD, Tweedy JH, Niederer SA, Peters NS, Vigmond EJ. Determinants of new wavefront locations in cholinergic atrial fibrillation. Europace 2018;20:III3–III15.

[11] Handa BS, Roney CH, Houston C, Qureshi NA, Li X, Pitcher DS, Chowdhury RA, Lim PB, Dupont E, Niederer SA, Cantwell CD, Peters NS, Ng FS. Analytical approaches for myocardial fibrillation signals. Comput Biol Med 2018;102:315–26.

**Supplementary Figures**

**Supplementary Figure 1: Study design.** A schematic of the chronology of the experiments performed. Refer to text in methodology section for details.


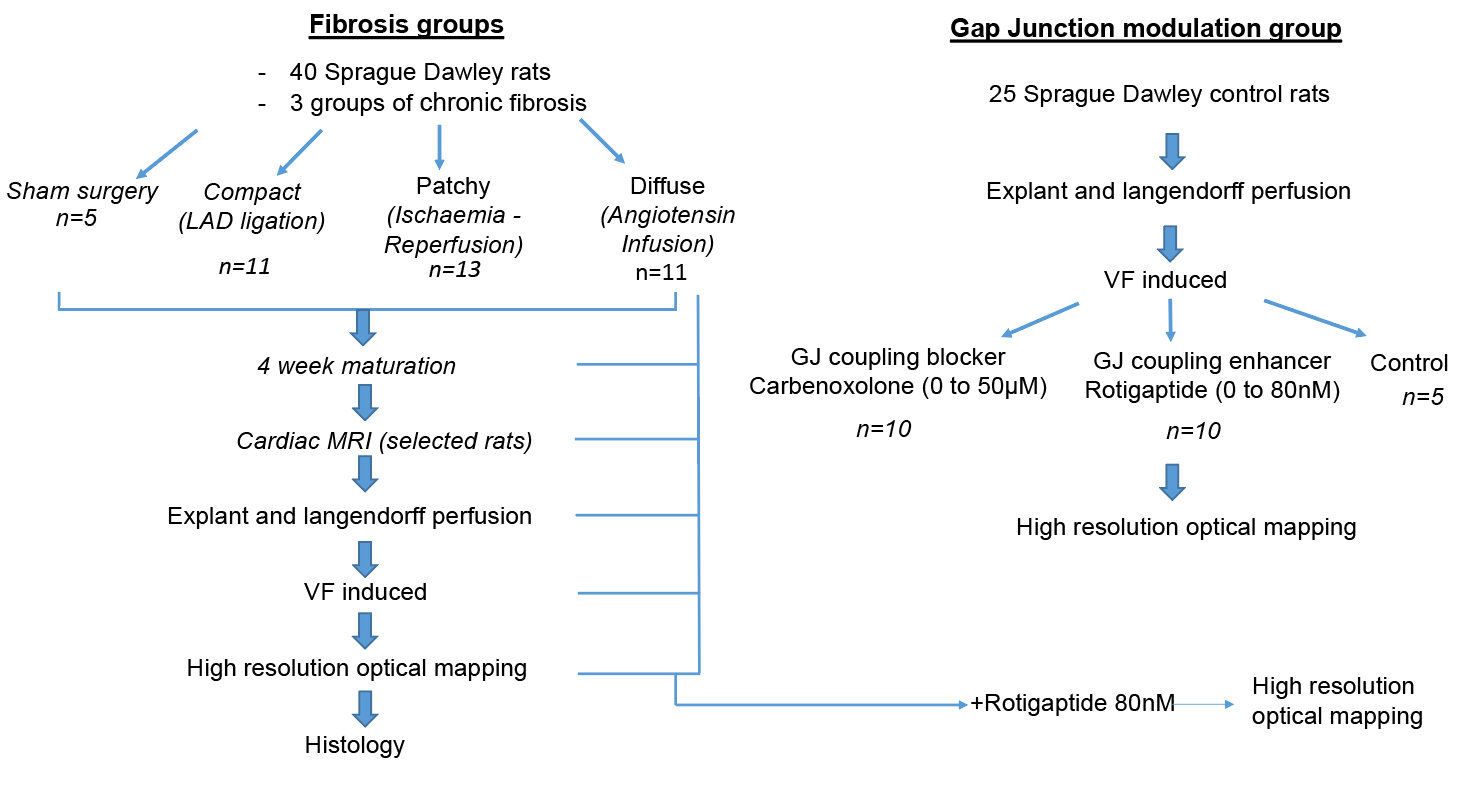


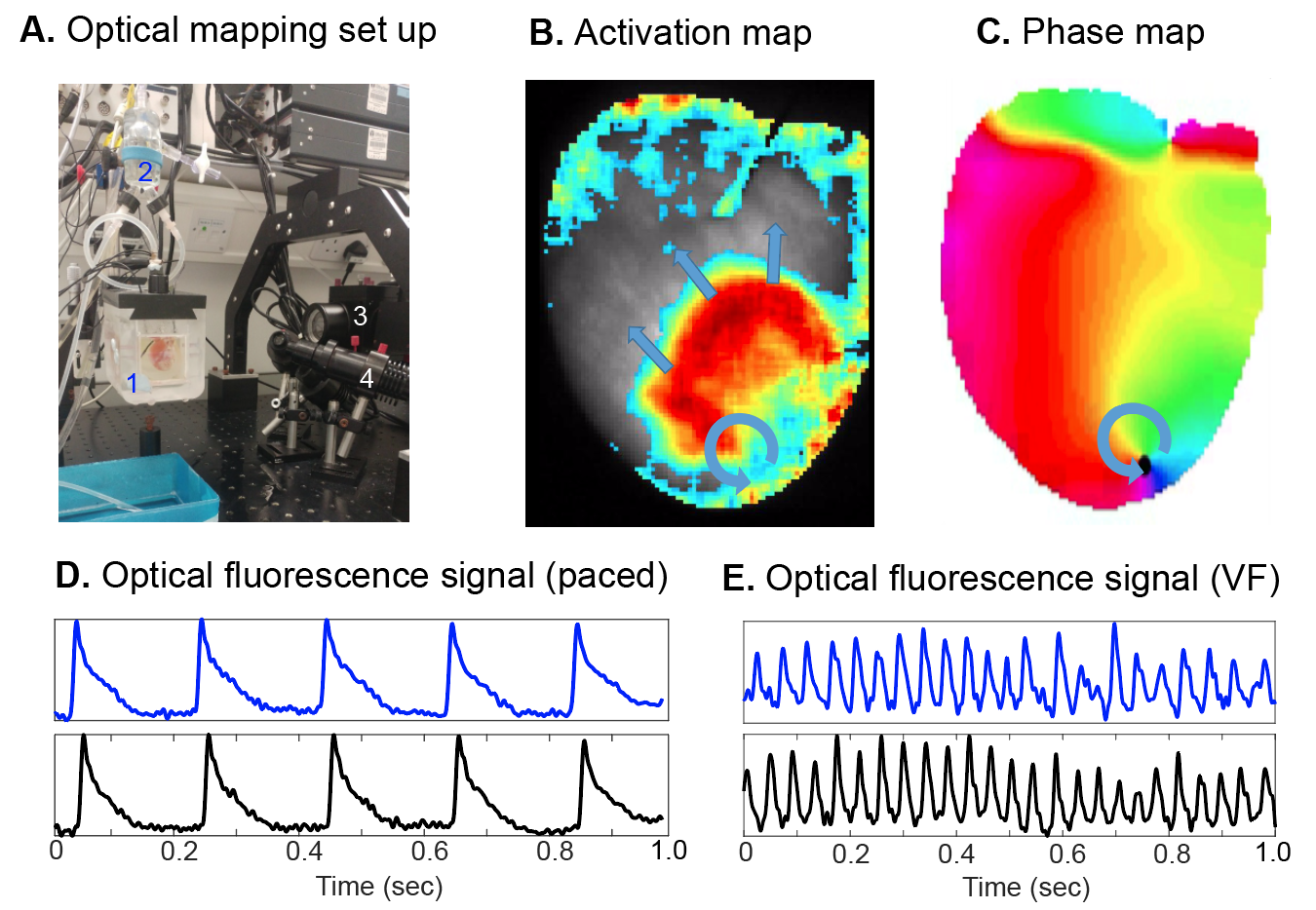
**Figure 2.** **Optical mapping data acquisition**. **(A)** Optical mapping camera set up for the study, 1 – perfused heart in a perspex chamber, 2 – Krebs-Henseleit perfusion system, 3 – CMOS camera, 4 – LED light (530nm wavelength). **(B)** Representative still of a flouresence snapshot showing a depolarising wavefront propogating from a RA (Supplementary methods video 1). **(C)** Representative still from the corresponding phase processed video with a tracked RA (black dot, Supplementary methods video 2). **(D)** Representative optical flouresence traces in a heart paced at a 200ms cycle length. **(E)** Representative optical flouresence traces in VF.

**Figure 3.** **Frequency dominance index.** A representative dominant frequency (DF) map in VF (left) and the corresponding global DF histogram (right). The FDI is defined as the largest organised DF area in the global fibrillatory spectrum relative to the total area of all regions with a defined DF.


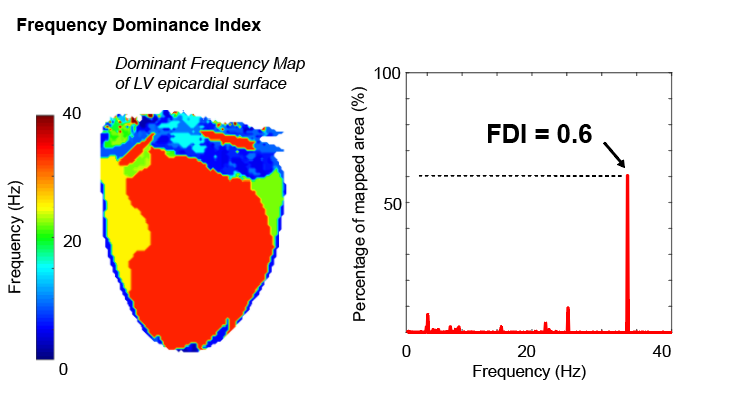


**Figure 4. Baseline Conduction Velocity and APD90.** Graphs showing the baseline conduction velocity (CV) and APD90 in response to differing pacing cycle length in (A) the gap junction groups at baseline and (B) differing fibrosis groups at baseline. Data from basal remote myocardium in sham (n=5), permanent MI (n=11), angiotensin infusion (n=11) and ischaemia reperfusions hearts (n=13) in (A) and rotigaptide 0nM (n=10) and carbenoxolone 0µM (n=10) hearts in (B). All values are reported as mean +/- standard deviation.


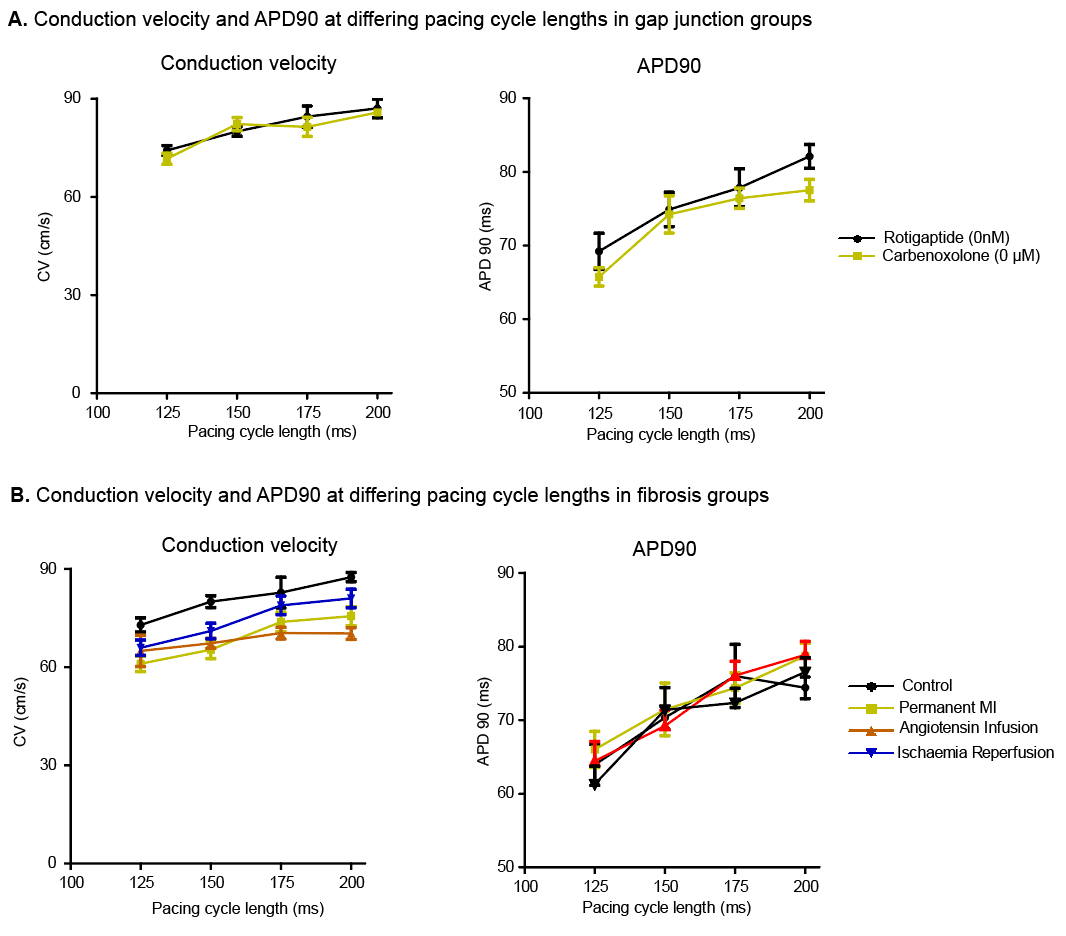


**Supplementary Figure 5. ECG dominant frequency (DF) response with GJ coupling.**  Representative graphs showing the DF power spectral density of ECGs in response (A) rotigaptide (RTG, 0-80nM) and (B) carbenoxolone (CBX, 0-50µM) corresponding to ECG in Figure 1A and 1B. The maximal DFs are annotated and PSD scale is normalised.


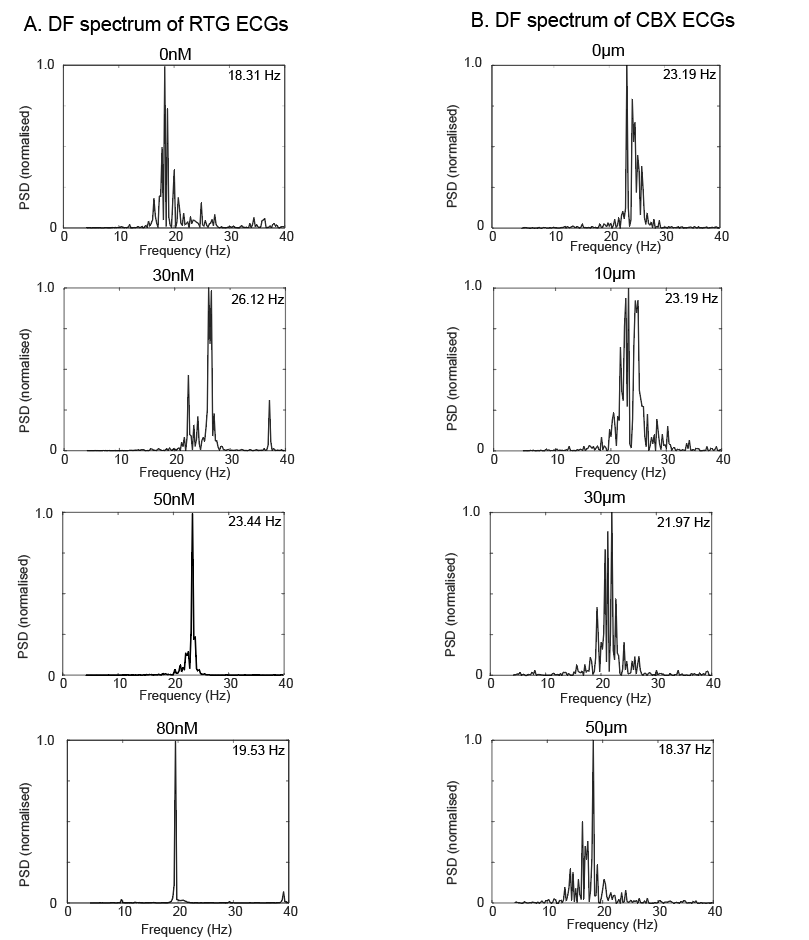


**Supplementary Figure 6.** Dominant frequency histograms in response to rotigaptide **(A)** and **(B)** carbenoxolone corresponding to dominant frequency maps in Figure 3.


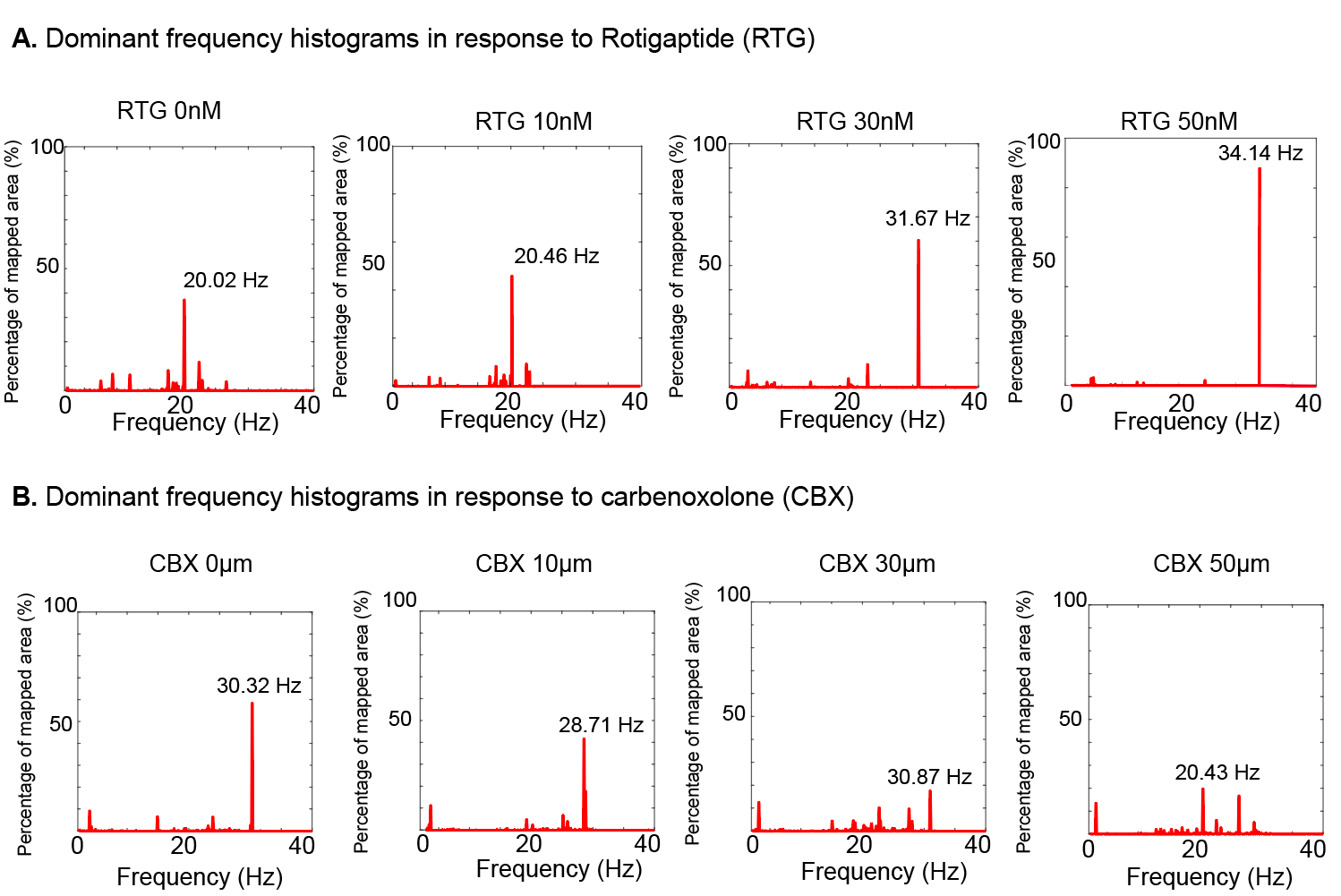


**Supplementary Figure 7. Fibrillatory mechanisms remain stable over time in the control group. (A)** Representative DF maps of VF, **(B)** FDI, **(C)** Representative RA heat map and **(D)** percentage of time VF is driven by RAs in the control group over 0, 10, 20 and 30 minutes. Data from place (n =5) treated hearts. Statistical analysis with repeated measures ANOVA, *post hoc* Bonferonni.


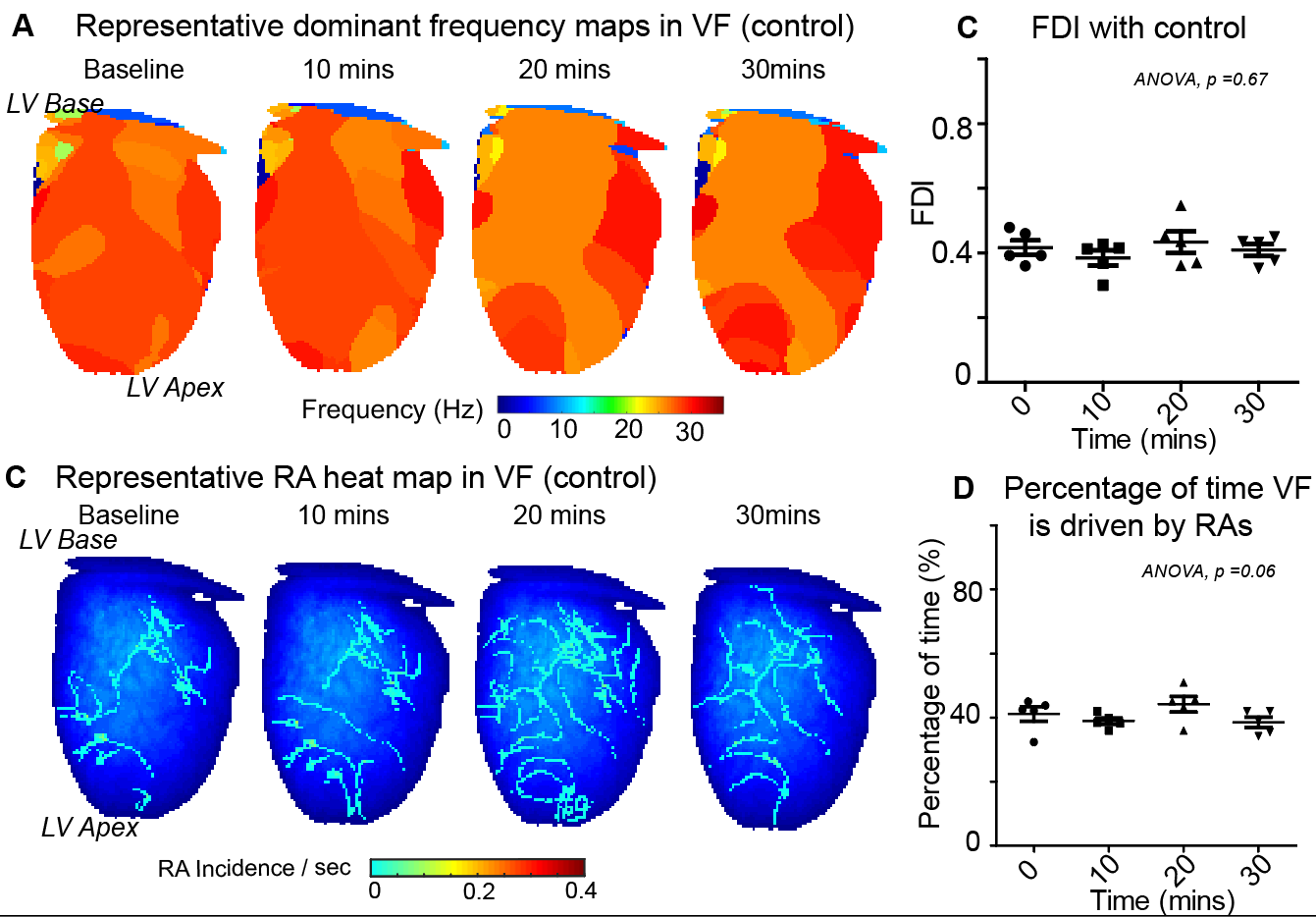


**Supplementary Figure 8. Fibrillatory mechanisms remain stable over time in the control group. (A)** Maximum duration for a single RA, **(B)** average rotations for all RAs, **(C)** Total number of RAs per second and **(D)** maximum rotations for a single RA in the control group (n=5) over 0,10,20 and 30 minutes. Data from control (n =5) treated hearts. Statistical analysis with repeated measures ANOVA, *post hoc* Bonferonni, p values are in comparison to baseline, *=p<0.05.


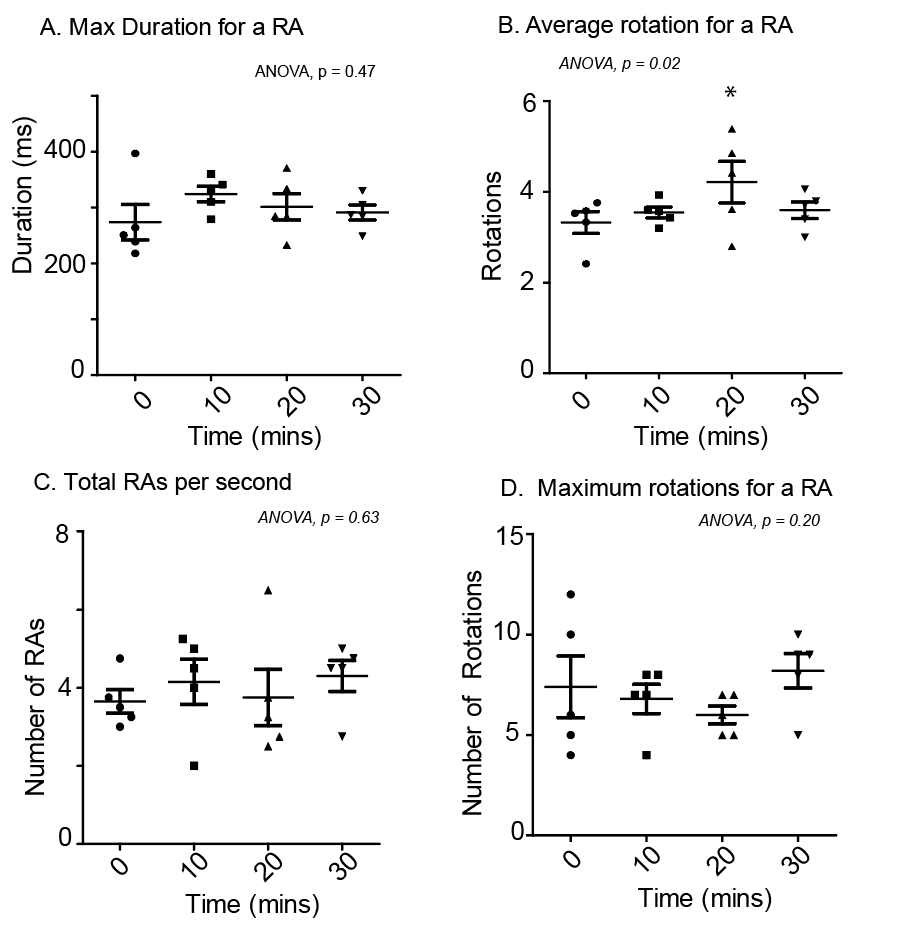


**Supplementary Figure 9. Enhanced coupling with rotigaptide stabilised rotational activities in fibrillation.** Increased maximum duration for a single RA **(A)** and increased average rotation per RA **(B)** in response to increasing rotigaptide concentration in VF. **(C)** Total number of RAs per second and **(D)** total duration of all tracked RAs per second of VF recordings in response to rotigaptide. Data from RTG (n =10) treated hearts. Statistical analysis with repeated measures ANOVA, *post hoc* Bonferonni, p values are in comparison to baseline, *=p<0.05, **=p<0.01, ***=p<0.001.


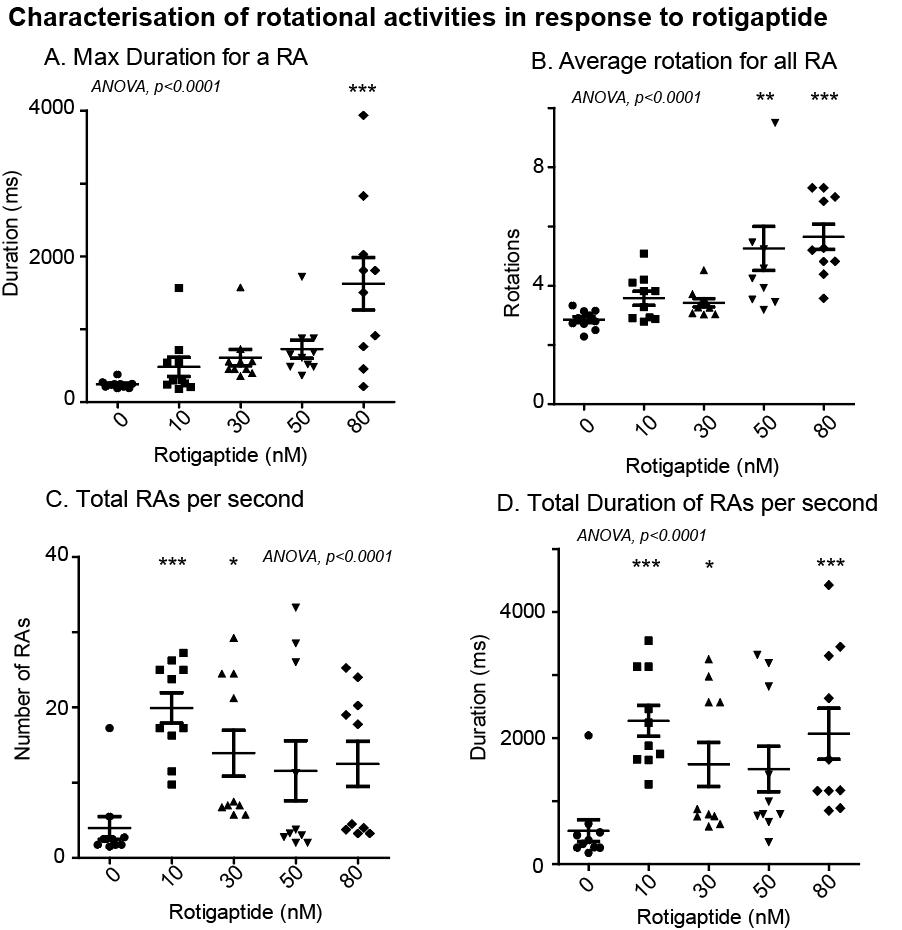


**Supplementary Figure 10. Gap junction uncoupling with carbenoxolone destabilises rotational activities in fibrillation.** Decreased maximum duration for a single RA **(A)** and decreased average rotation per RA **(B)** in response to increasing carbenoxolone concentration in VF. **(C)** Total number of RAs per second and **(D)** total duration of all tracked RAs per second of VF recordings in response to carbenoxolone. Data from CBX (n =10) treated hearts. Statistical analysis with repeated measures ANOVA, *post hoc* Bonferonni, p values are in comparison to baseline, *=p<0.05, **=p<0.01, ***=p<0.001.


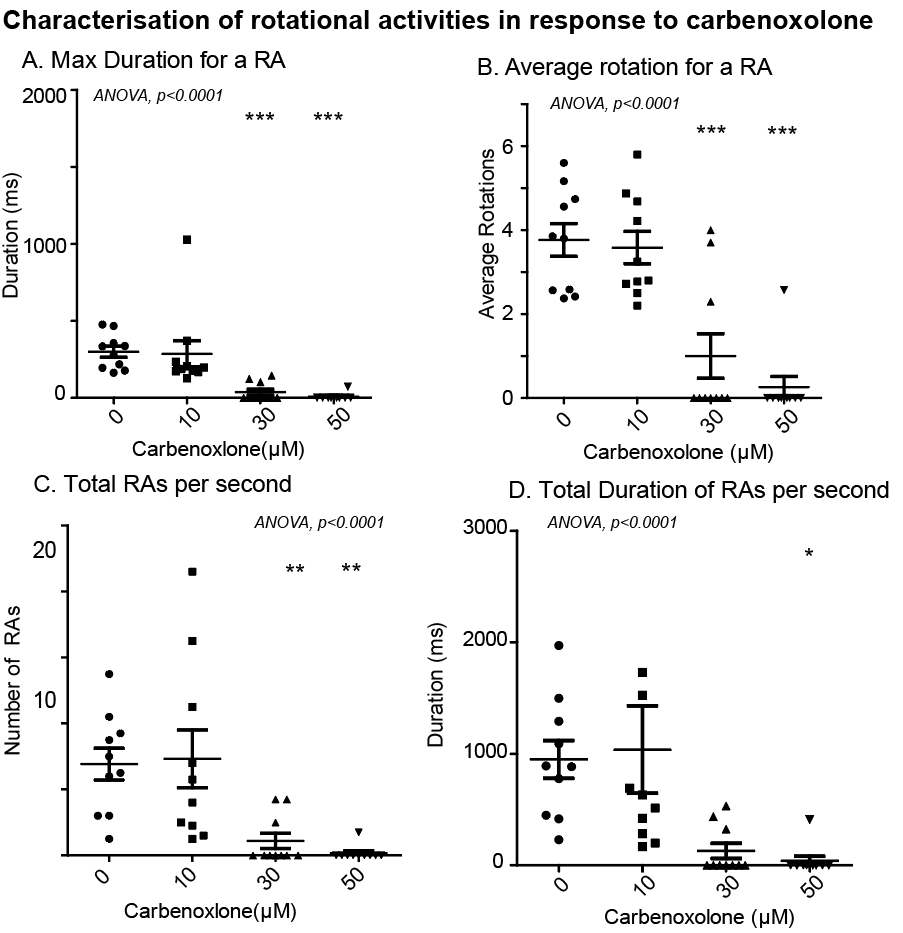


**Supplementary Figure 11. ECG dominant frequency (DF) response with fibrosis.**  Representative graphs showing the DF power spectral density (PSD) of ECGs in differing fibrosis models corresponding to ECGs in Figure 4A. The maximal DFs are annotated and PSD is normalised.


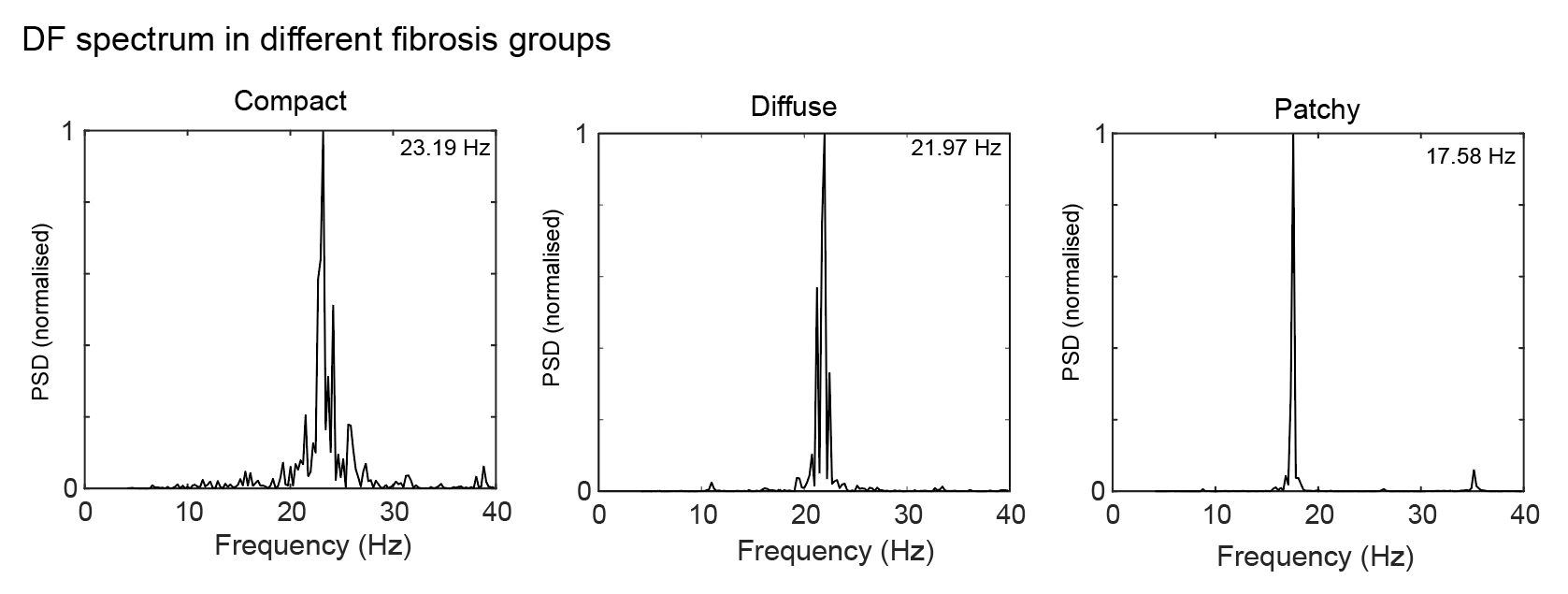


**Supplementary Figure 12. Arrhythmia provocation scoring. (A)** A representative ECG trace from an extra stimulus protocol with induction of sustained VF. An 8-beat S1 drive train of 100ms cycle length was followed by progressively earlier S2, S3 and S4 stimuli. **(B)** A representative ECG trace from a # protocol with induction of sustained VF. A 20 beat train of pacing stimuli delivered at a cycle length of 60ms. **(C)** Arrhythmia provocation scores (APS) in differing fibrosis models. Data from sham surgery (n=5), compact (n=11), diffuse (n=11) and patchy (n=13) fibrosis hearts. Statistical analysis with ANOVA, post hoc Bonferonni multiple comparisons test, **=p<0.01, ***=p<0.001.


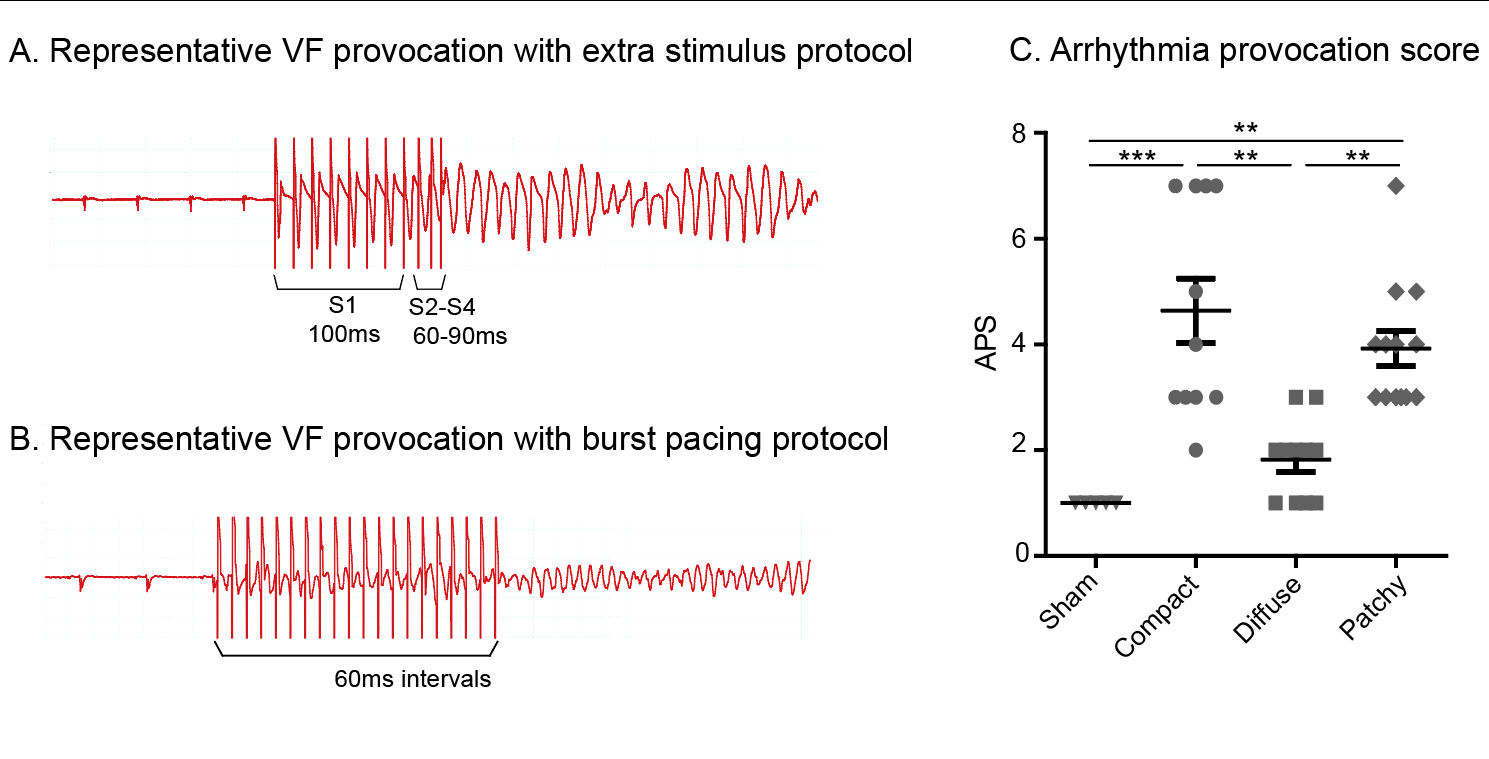


**Supplementary Figure 13. MRI characterisation of differing fibrosis groups.** Representative ventricular axial sections showing traced fibrosis as characterised by LGE (white transmural enhancement) with corresponding American Heart Association 17 segment model of fibrosis quantification per segment in compact **(A)**, diffuse **(B)** and patchy **(C)** fibrosis groups. **(D)** Total quantity of fibrosis in differing fibrosis model as characterised by percentage LGE. Data from select sham surgery (n=3), compact (n=5), patchy (n=5) and diffuse (n=5) fibrosis hearts. Statistical analysis with repeated measures ANOVA, *post hoc* Bonferonni, p values are in comparison to baseline, *=p<0.05, **=p<0.01, ***=p<0.001.


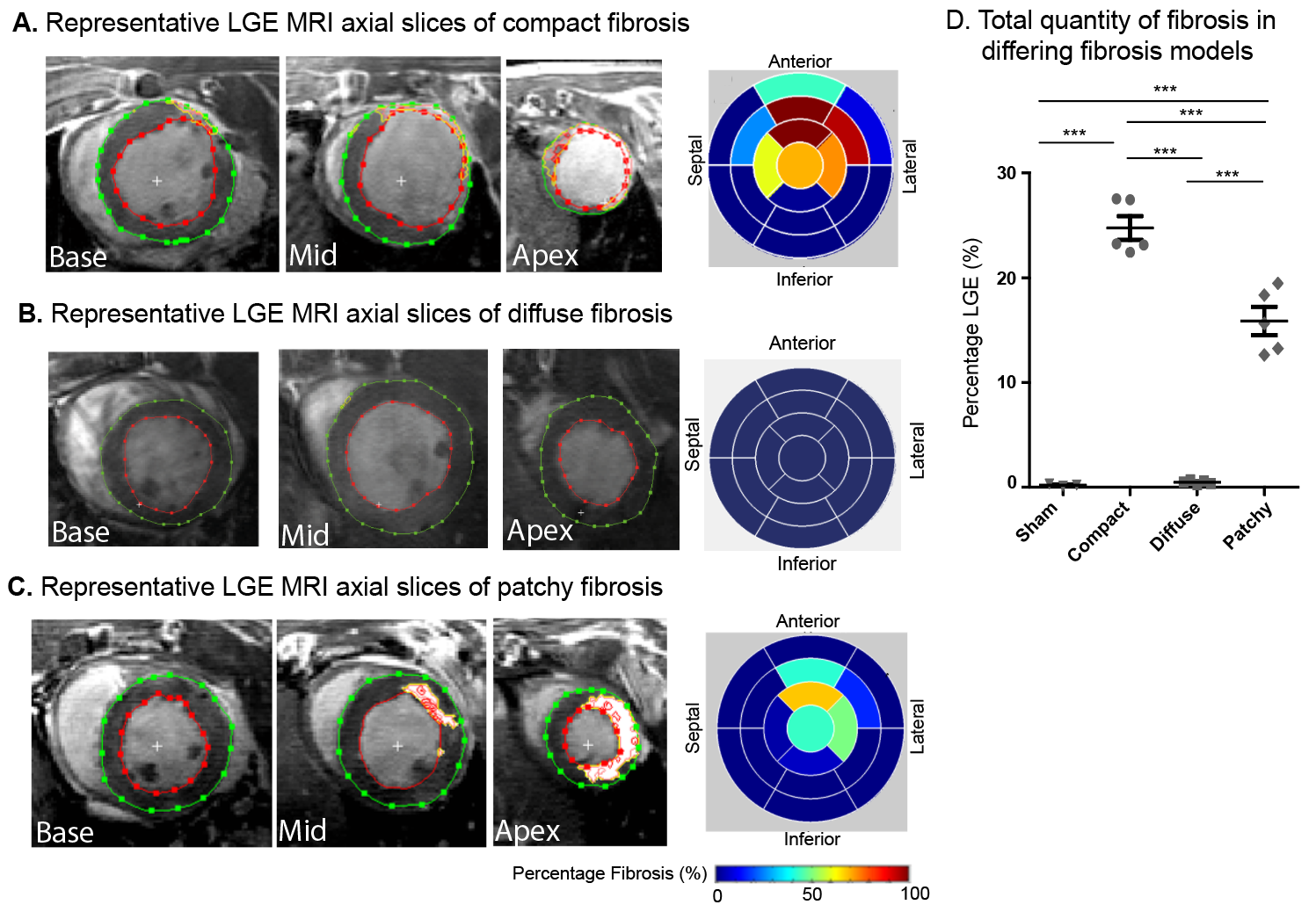


**Supplementary Figure 14:** **(A)** APD 90 and **(B)** APD 90 dispersion in the differing fibrosis groups (paced at 200ms cycle length). R – remote, IB – infarct border. Data from sham (n=5), permanent MI (n=11), angiotensin (500ng/kg/min, n=11) and IR (20 mins ischaemia, n=13) hearts. Statistical analysis with ANOVA *post hoc* Bonferonni multiple comparisons test, ***=p<0.001.


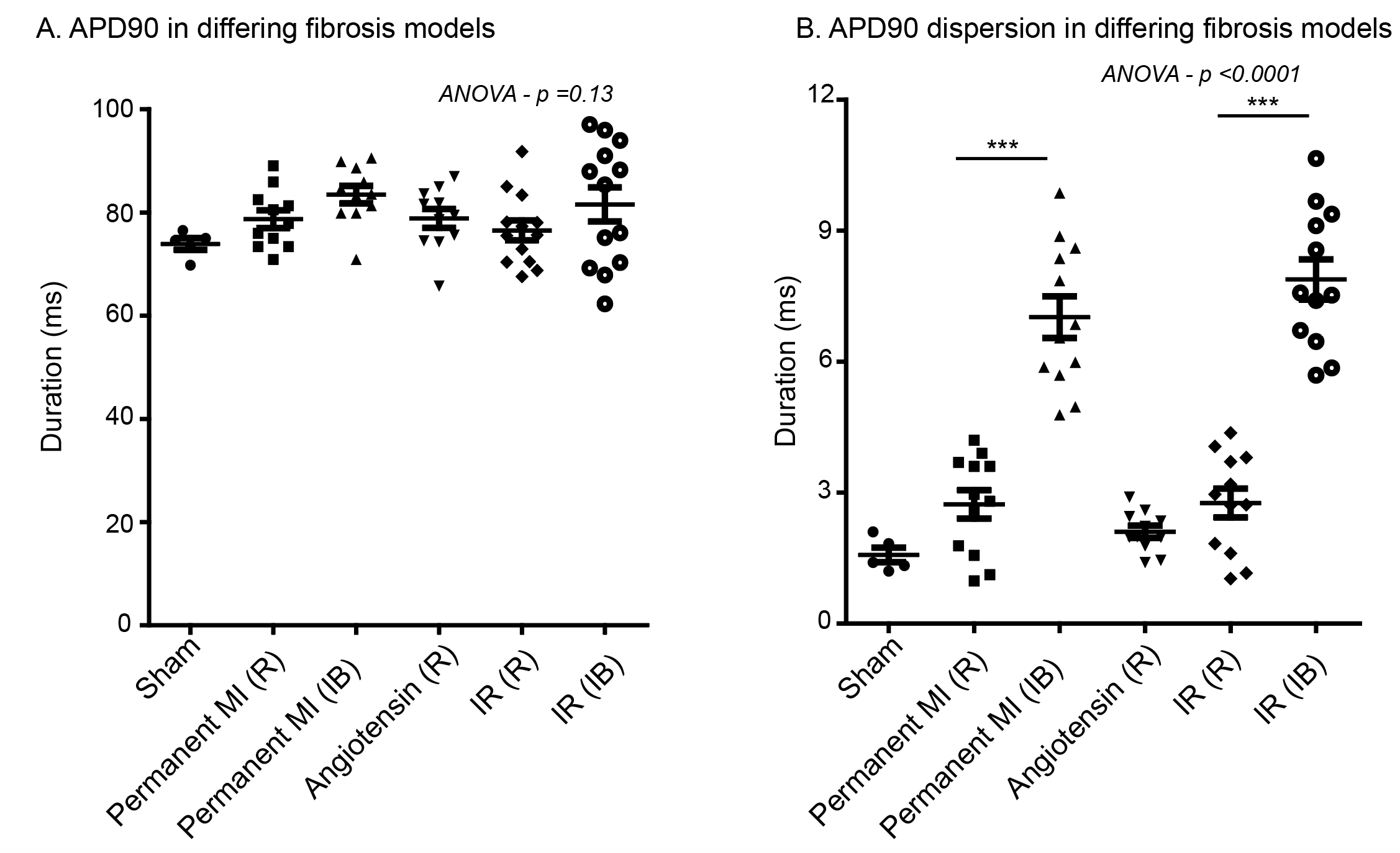


**Supplementary Figure 15. Patchy fibrosis stabilises rotational activities in fibrillation.** Maximum duration for a single RA **(A)**, average rotation per RA **(B)**, total RAs per second **(C)** and total duration of all tracked RAs per second **(D)** in fibrillation is the highest in the patchy fibrosis group comparative to diffuse and compact fibrosis. Data from sham surgery (n=5), compact (n=11), diffuse (n=11) and patchy (n=13) fibrosis hearts. Statistical analysis with ANOVA, post hoc Bonferonni multiple comparisons test, *=p<0.05, **=p<0.01, ***=p<0.001.


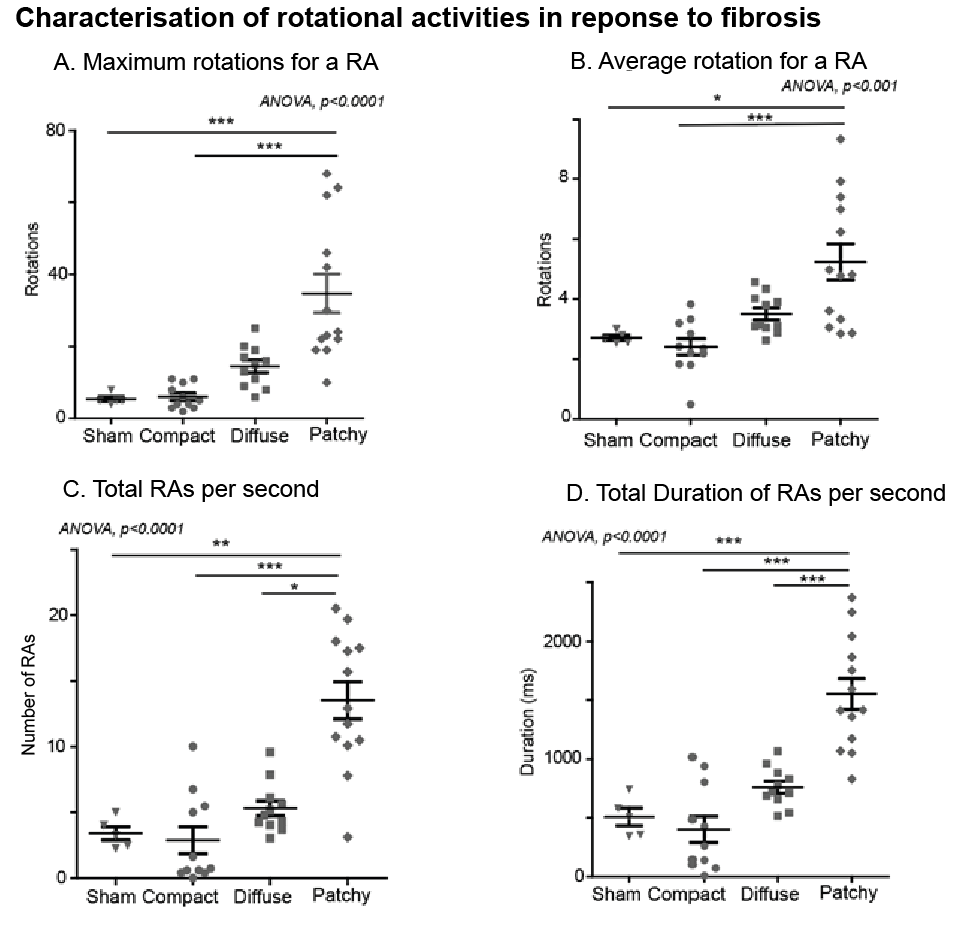


**Supplementary Figure 16.** Dominant frequency histograms in response to differing fibrosis models corresponding to DF maps in Figure 6A.


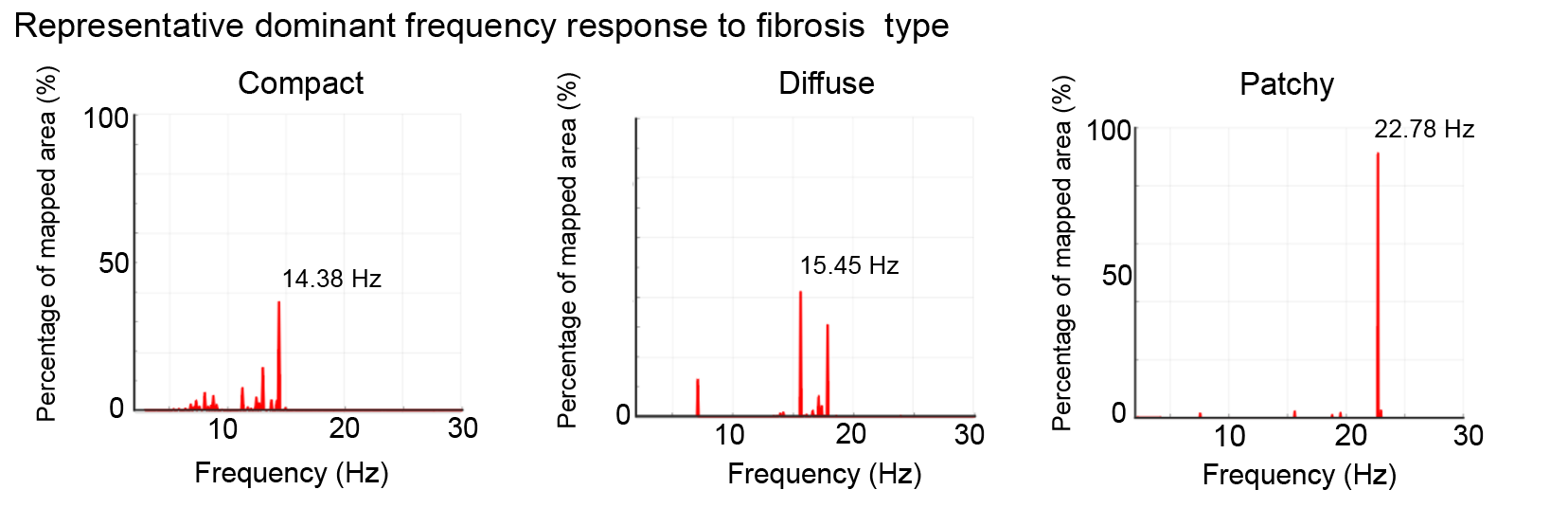


**Supplementary Figure 17.** **Enhanced GJ coupling in diffuse fibrosis hearts can organise and terminate VF.** **(A)** Sample ECG traces from diffuse fibrosis hearts that cardioverted from VF to sinus rhythm after RTG 80nM infusion with **(B)** representative DF maps pre and post RTG infusion with corresponding increase in FDI response with RTG. Data from diffuse (n=11) fibrosis hearts. Statistical analysis with student t-test, **=p<0.01.


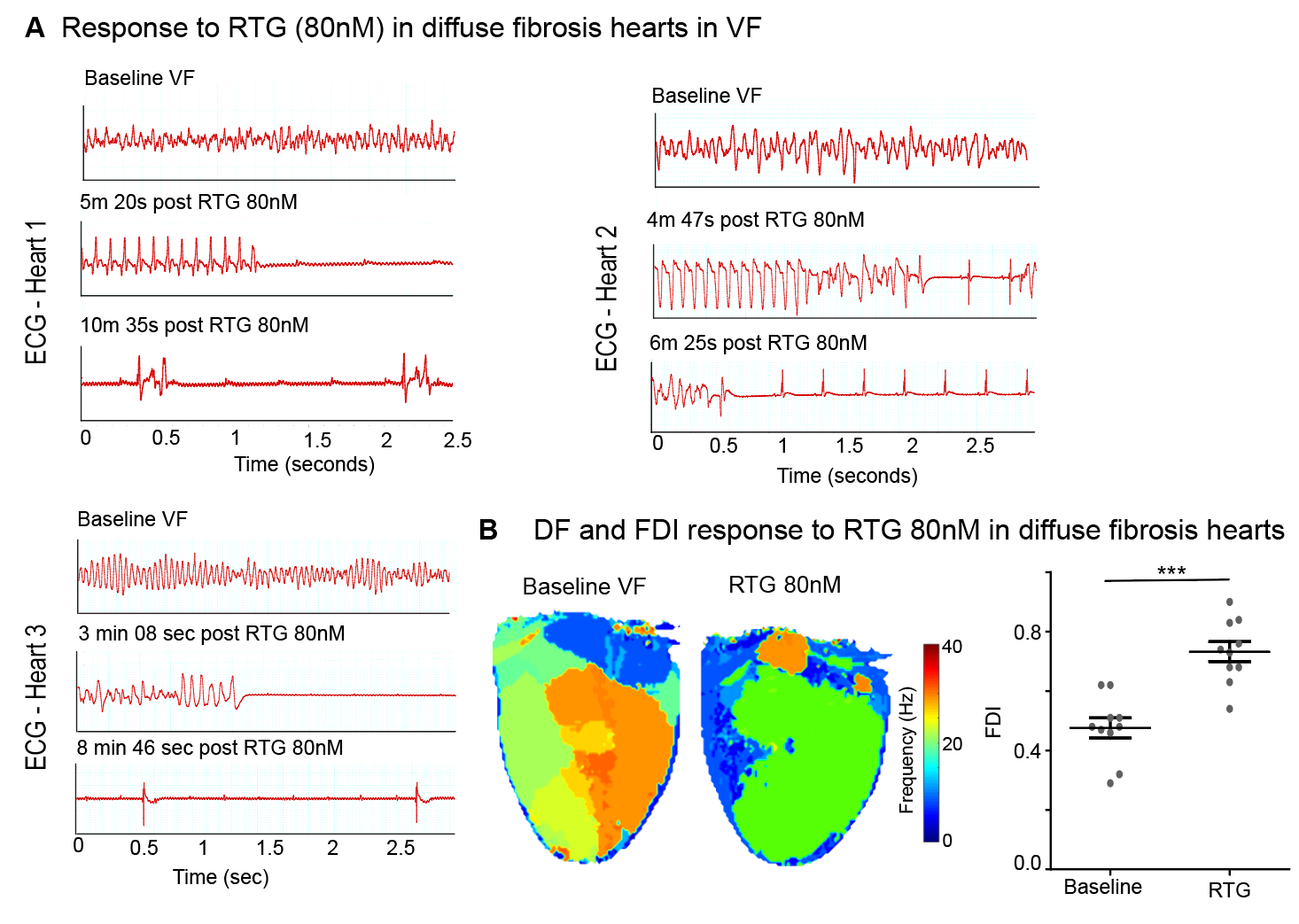


**Supplementary Videos**

Attached are representative supplementary phase processed video of VF with rotational activity tracking. A ≥2 rotations threshold is utilised to define a rotational activity. The black dots track clockwise rotating rotational activities and white white anti-clockwise rotating rotational activities. Video 1 to 3 are in control hearts; **Video 1** - Baseline VF, **Video 2** – VF after infusion of 50µM carbenoxolone and **Video** 3 - VF after infusion of 80nM rotigaptide. Video 4-6 are VF in fibrotic hearts; **Video 4** – compact fibrosis, **Video** 5 – diffuse fibrosis and **Video 6** – patchy fibrosis. Recordings are of LV anterior epicardial surface. Video playback is slowed to 30 milliseconds per second. Transition in phase corresponding to videos as below:


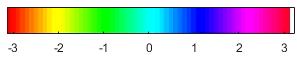

Supplement: cvaa141_Supplementary_Data [file cvaa141_supplementary_data.zip › Supplement R2 CLEAN.docx]
